# Flavonoids: A treasure house of prospective pharmacological potentials

**DOI:** 10.1016/j.heliyon.2024.e27533

**Published:** 2024-03-09

**Authors:** Hasin Hasnat, Suriya Akter Shompa, Md. Mirazul Islam, Safaet Alam, Fahmida Tasnim Richi, Nazim Uddin Emon, Sania Ashrafi, Nazim Uddin Ahmed, Md. Nafees Rahman Chowdhury, Nour Fatema, Md. Sakhawat Hossain, Avoy Ghosh, Firoj Ahmed

**Affiliations:** a Department of Pharmacy, State University of Bangladesh, 77 Satmasjid Road, Dhanmondi, Dhaka, 1207, Bangladesh; b Department of Pharmaceutical Chemistry, Faculty of Pharmacy, University of Dhaka, Dhaka, 1000, Bangladesh; c Drugs and Toxins Research Division, BCSIR Laboratories Rajshahi, Bangladesh Council of Scientific and Industrial Research, Rajshahi, 6206, Bangladesh; d Department of Pharmacy, Faculty of Science and Engineering, International Islamic University Chittagong, Chittagong, 4318, Bangladesh; e Department of Pharmacy, Faculty of Pharmacy, University of Dhaka, Dhaka, 1000, Bangladesh; f Pharmaceutical Sciences Research Division, BCSIR Dhaka Laboratories, Bangladesh Council of Scientific and Industrial Research (BCSIR), Dr. Qudrat-I-Khuda Road, Dhanmondi, Dhaka, 1205, Bangladesh; g Department of Microbiology, Stamford University Bangladesh, Dhaka, 1217, Bangladesh

**Keywords:** Flavonoids, Apigenin, Quercetin, Kaempferol, Galangin, Naringenin, Hesperetin, Myricetin, Antioxidants, Flavonol, Flavone, Flavonone

## Abstract

Flavonoids are organic compounds characterized by a range of phenolic structures, which are abundantly present in various natural sources such as fruits, vegetables, cereals, bark, roots, stems, flowers, tea, and wine. The health advantages of these natural substances are renowned, and initiatives are being taken to extract the flavonoids. Apigenin, galangin, hesperetin, kaempferol, myricetin, naringenin, and quercetin are the seven most common compounds belonging to this class. A thorough analysis of bibliographic records from reliable sources including Google Scholar, Web of Science, PubMed, ScienceDirect, MEDLINE, and others was done to learn more about the biological activities of these flavonoids. These flavonoids appear to have promising anti-diabetic, anti-inflammatory, antibacterial, antioxidant, antiviral, cytotoxic, and lipid-lowering activities, according to evidence from *in vitro*, *in vivo*, and clinical research. The review contains recent trends, therapeutical interventions, and futuristic aspects of flavonoids to treat several diseases like diabetes, inflammation, bacterial and viral infections, cancers, and cardiovascular diseases. However, this manuscript should be handy in future drug discovery. Despite these encouraging findings, a notable gap exists in clinical research, hindering a comprehensive understanding of the effects of flavonoids at both high and low concentrations on human health. Future investigations should prioritize exploring bioavailability, given the potential for high inter-individual variation. As a starting point for further study on these flavonoids, this review paper may promote identifying and creating innovative therapeutic uses.

## Graphical abstract

**Figure:**
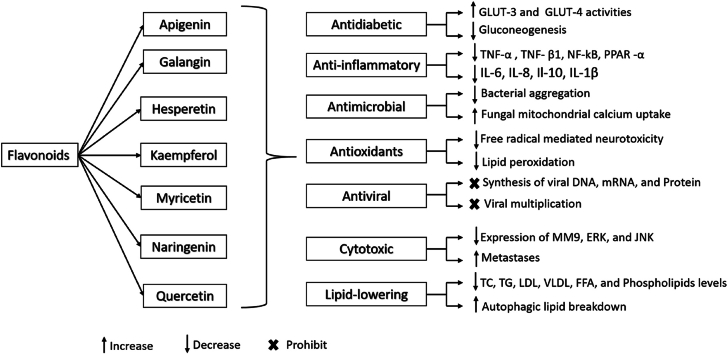

## 1 Introduction

Plants are a constant source of essential medicinal compounds [1]. They have served as a source of medications to cure a range of conditions since ancient times. People predicted the therapeutic benefits of medicinal plants before the creation of contemporary therapies [2]. The use of traditional and unconventional methods to treat various illness conditions is widespread worldwide [3]. Even in the modern period, plants are still a possible source of therapeutics for serious illnesses like cancer, oxidative stress, diarrhea, depression, fever, and thrombosis [4]. However, recently, natural plant-based products have attracted interest as a significant source of novel, secure, and powerful secondary bioactive metabolites with medicinal potential [5]. Around the world, 80% of people utilize plant-based medicines as part of their key healthcare, according to the World Health Organization [6]. Despite the fact that many synthetic medicines have been made available for purchase on the market to treat illness, they are afflicted by a number of dangerous adverse effects. On the other hand, medications made from plants have stronger therapeutic effects and fewer negative effects [7]. It is anticipated that plant-based medicines will make up to 25% of all drugs in developed nations like America, in the meantime, they will make up around 80% of all drugs in rapidly growing nations like India and China [6].

Polyphenols are complex organic compounds present in a variety of plant-based regimens. The phenolic ring, which is commonly separated into phenolic acids and phenolic alcohols, is the basic building component of polyphenols. There are two basic categories of polyphenols: firstly, flavonoids and secondly non-flavonoids (for example lignans, stilbenes, phenolic acids, non-phenolic metabolites, and other polyphenols), which together make up more than 8000 different chemicals. Different kinds of polyphenols can be recognized based on how strong the phenolic ring is [8,9].

Flavonoids are naturally produced compounds that are derived from plants and are found in many plant parts. They are a class of widely dispersed low-molecular-weight phenolic molecules. These are among the most unique classes of molecules present in advanced plants. Flavonoids are necessary for vegetables' growth and self-defence against plaques [10,11]. They are also referred to as “dietary flavonoids” due to the fact that they can be found in a variety of plant-based aliments and drinks, including fruits, vegetables, tea, chocolate, and wine [12]. The basic carbon skeleton of a flavonoid is made up of the flavan nucleus, which has 15 carbons arranged in two aromatic rings connected by a three-carbon bridge and forming a diphenyl propane structure (C6-C3-C6) that might or might not be a part of the third ring [13]. Flavonoids are divided into six main categories, namely flavones (such as apigenin and luteolin), flavonols (such as quercetin and myricetin), flavanones (such as naringenin and hesperidin), catechins or flavanols (such as epicatechin and gallocatechin), anthocyanidins (such as cyanidin and pelargonidin), and isoflavones (such as genistein and daidzein) [14]. However, they can be found as aglycones, which lack bound sugars, or as glycosides, which have bound sugars (glycosyl groups) [14]. They have been said to have anti-inflammatory, antioxidant, antibacterial, antiviral, antiallergic, cytotoxic, and anticancer activities. They are also used to treat neurological illnesses and have a vasodilatory effect. Additionally, flavonoids have been shown to reduce the enzyme activity of cyclooxygenase, lipoxygenase, lipid peroxidation, platelet aggregation, capillary permeability, and fragility. However, they block a wide range of enzymes, including hydrolases, hyaluronidase, alkaline phosphatase (ALP), arylsulphatase, cAMP phosphodiesterase, lipase, and alpha-glucosidase kinase [15].

**Fig. 1:**
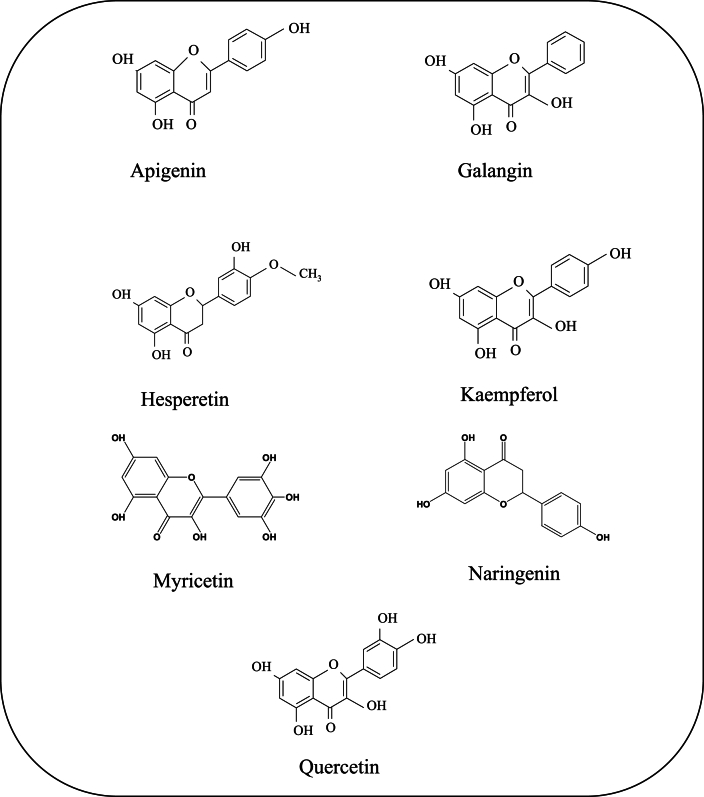
Chemical structures of important bioactive flavonoids.

The biological effects of flavonoids derived from dietary sources are thoroughly examined in this review work, with a special emphasis on seven of the most prevalent ones: apigenin, galangin, hesperetin, kaempferol, myricetin, naringenin, and quercetin. The focus of the investigation is on their prospective health advantages, which include lipid-lowering, antidiabetic, anti-inflammatory, antimicrobial, antioxidant, and antiviral characteristics. This review work is expected to provide a greater understanding of the many therapeutic potentials of these particular flavonoids as well as their significance in advancing human health by doing so. While numerous studies, spanning in *vivo, in vitro, in silico*, and clinical investigations, have explored various aspects, these findings have not been synthesized into a cohesive article. This article uniquely compiles and integrates these diverse findings into a single comprehensive resource. The structures of these substances are displayed in Fig. 1. Additionally, some common source of these compounds is shown in Table 1.

**Table 1:** Flavonoids and their plant sources along with isolation techniques.

Compound	Plant	Family	Plant material	Extract	Isolation technique	Reference
Apigenin	*Portulaca oleracea* L.	Portulacaceae	Aerial part	Ethanol	TLC, IR spectra, LC-MS, NMR, and HPLC	[16]
	*Matricaria chamomilla* L.	Asteraceae	Flower	Methanol	HPLC–MS–MS	[17]
	*Aster yomena* (Kitam.) Honda	Asteraceae	Aerial part	Methanol	SGCC, HPLC, 1H NMR, 13C NMR	[18]
	*Daphne genkwa* Siebold et Zuccarini	Thymelaeaceae	Flower	Ethanol	CC, RP-HPLC	[19]
	*Cajanus cajan* (L.) Millsp.	Fabaceae	Root	Ethanol	RP-HPLC	[20]
	*Chrysanthemum morifolium*	Asteraceae	Flower	Aqueous	HPLC, Q-TOF/MS	[21]
	*Cajanus cajan* (L.) Millsp	Fabaceae	Leaves	Aqueous	RTCC, RP-HPLC	[22]
	*Chamomilla matricaria* L.	Asteraceae	Flowers	Aqueous	TLC,CC,HPTLC-UV/VIS	[23]
	*Perilla frutescens* (L.) Britton	Lamiaceae	Seed	Methanol	EI-MS, NMR	[24]
	*Matricaria chamomilla* L.	Asteraceae	Flower	Methanol	LC/MS, HPLC,LC/MS/MS,NMR	[25]
	*Gentiana veitchiorum* Hemsl	Gentianaceae	Flower	Methanol	HPLC–MS/MS	[26]
	*Justicia gendarussa*Burm. f.	Acanthaceae	Roots	Methanol	NMR,TLC,CC	[27]
Galangin	*Alpinia officinarum Hance*	Zingiberaceae	Rhizome	Chloroform and methanol	UV spectr,IR spectra,NMR	[28]
	*Helichrysum aureonitens*	Asteraceae	Aerial part	Acetone	CC,HPLC,TLC, UV-VIS	[29]
	*Alpinia officinarum* Hance	Zingiberaceae	Rhizome	Methanol	RP-HPLC,UV, IR spectra, MS, 1H-NMR, 13C-NMR	[30]
	*Alpinia calcarata (Haw.) Roscoe*	Zingiberaceae	Rhizome	Chloroform	CC,TLC, NMR	[31]
	*Populus deltoides*Bartram ex Marsh.	Salicaceae	Bud exudates		GC-MS	[32]
	*Populus fremontii* S. Watson	Salicaceae	Bud exudates		GC-MS	[32]
	*Populus sargentii*	Salicaceae	Bud exudates		GC-MS	[32]
	*Populus wislizenii*	Salicaceae	Bud exudates		GC-MS	[32]
Hesperetin	*Trifolium alexandrinum* Linn.	Fabaceae	Aerial part	Ethyl acetate	CE, SGCC, HPLC	[33]
	*Cordia sebestena*	Boraginaceae	Flowers	Methanol	CC,FT-IR,LC-MS,H-NMR,C-NMR,HSQC,HMBC	[34]
	*Origanum majorana* L.	Lamiaceae	Aerial part	Hexane and ethyl acetate	TLC,HPLC-TOF/MS,UV spectra,IR spectra,NMR	[35]
	*Citrus aurantium*	Rutaceae	Fruits	DMSO	LC, NMR,HPLC	[36]
	*Citrus sinensis*	Rutaceae	Peels	Petroleum ether	UV, IR, NMR	[37]
	*Citrus aurantiifolia*	Rutaceae	Fruits	Methanol	FT-IR,LC-MS,HPLC,MS	[38]
	*Clerodendrum petasites S*	Lamiaceae	Root	Ethanol	HPLC	[39]
	*Aurantii fructus*	Rutaceae	Whole plant	Ethanol	ESI-Q-TOF-MS, GC-MS, HPLC	[40]
	*Backhousia citriodora*	Myrtaceae	Leaves	Acetone	HPLC	[41]
	*Citrus aurantium* L.	Rutaceae	Dried fruits	Ethanol	RT-PCR	[42]
Kaempferol	*Polygonum tinctorium Lour.*	Polygonaceae	Aerial part	Ethyl acetate	NMR	[43]
	*Crocus sativus*	Iridaceae	Flowers	Methanol	UV	[44]
	*Acacia nilotica (L.) Willd. ex Delile*	Leguminosae	Bark	Methanol	NMR and MS	[45]
	*Crocus sativus*	Iridaceae	Leaves	Formic acid,Methanol,water	NMR, HPLC,HPLC-ESI-MS/MS	[46]
	*Eruca sativa*	Brassicaceae	Seeds	Hydro-alcoholic	NMR	[47]
	*Lagenaria siceraria*	Cucurbitaceae	Fruits	Methanol	NMR	[48]
	*Cedrela odorata* L.	Meliaceae	Leaves	Methanol	1H and 13C NMR	[49]
	*Rubus idaeus*	Rosaceae	Fruits	Methanol	HPLC	[50]
	*Moringa oleifera*	Moringaceae	Leaves	Hydro-alcoholic	CC,LC-MS,HPLC	[51]
	*Dodonaea viscosa* var. *angustifolia*	Sapindaceae	Leaves	Methyl ether	1H and 13C NMR, MS	[52]
Myricetin	*Myrica rubra* Sieb. Et zucc	Myricaceae	Leaves	Ethanol	Polyamide column	[53]
	*Parrotia persica*	Hamamelidaceae	Leaves	Ethyl acetate	HPLC, NMR	[54]
	*Hypericum afrum* Lam.	Hypericaceae	Aerial part	Chloroform	NMR,HR-ESI–MS	[55]
	*Guiera senegalensis*	Combretaceae	Leaves	Ethanol	1 H NMR, 13C NMR and 2D-NMR, CC, HPLC.	[56]
	*Davilla elliptica*St.-Hil	Dilleniaceae	Aerial part	Ethanol	HPLC, NMR	[57]
	*Davilla elliptica* St.-Hil	Dilleniaceae	Aerial part	Ethanol	FT-IT, UV, HPLC, NMR	[58]
	*Limonium sinuatum*	Plumbaginaceae	Flowers	Methanol	CC, TLC, UV, MS	[59]
	*Murraya paniculata*	Rutaceae	Leaves	Chloroform	NMR,UV	[60]
	*Rhodomyrtus tomentosa*	Myrtacea	Fruits	Ethyl acetate	HPLC, NMR,MS	[61]
	*Myrica esculenta* Buch. Ham. Ex d. Don	Myricaceae	Bark	Methanol and aqueous	CC, UV	[62]
	*Acacia confusa*	Leguminosae	Leaves	methanolic	HPLC, 13C and 1H NMR, COSY, HMQC and HMBC	[63]
Naringenin	*Mentha aquatica*	Lamiaceae	Aerial parts	Ethanol	1H, 13C and 13C-DEPT NMR and optical rotation.	[64]
	*Citrus junos*	Rutaceae	Fruits	Methanol	1H and 13CNMR, EI-MS	[65]
	*Carissa carandas* L.	Apocynaceae	Leaves	Methanol	UPLC-MS/MS, 1H-NMR, 13C-NMR COSY, HMQC, HMBC and ESIMS.	[66]
	*Citrus grandis*	Rutaceae	Peels	Methanol	NMR, HPLC,UV,FT-IR	[67]
	*Citrus wilsonii*	Rutaceae	fruits	Aqueous	UPLC-MS/MS.	[68]
	*Rheedia gardneriana*	Clusiaceae	Leaves	Ethyl acetate	TLC, 1H-NMR, 13C-NMR	[69]
	*Cyclopia genistoides*	Fabaceae	Shoots	Ethanol	PR-HPLC, GC-MS, 1H-NMR, 13C-NMR.	[70]
	*Citrus reticulata*	Rutaceae	Peel	Methanoli	UV-spectroscopy, NMR, LC-MS	[71]
	*Nardostachys jatamansi*	Caprifoliaceae	Roots		1H and 13C-NMR	[72]
	*Nymphaea mexicana* Zucc.	Nymphaeaceae	Shoots	DCM	CC, FT-IR, 1H-NMR 13C-NMR.	[73]
	*Eleocharis dulcis*	Cyperaceae	Fruits	Ethyl acetate	HPLC, 1H-NMR	[74]
Quercetin	*Trifolium alexandrinum* Linn.	Fabaceae	Aerial part	Ethyl acetate	CE, SGCC, HPLC	[33]
	*Aesculus indica*	Sapindaceae	Fruits	Ethyl acetate	HPLC,NMR,FTIR	[75]
	*Nelumbo nucifera*	Nelumbonaceae	leaves	Butanol, ethanol	UV, EIMS, and NMR	[76]
	*Allium cepa*	Amaryllidaceae	Onion dry skin	Methanol	HPLC-MS/MS	[77]
	*Psidium guajava*	Myrtaceae	Leaves	Methanol	HPLC	[78]
	*Lagenaria siceraria*	Cucurbitaceae	Fruits	Ethyl acetate	HPLC, Co-TLC, UV and FTIR	[79]
	*Phaseolus vulgaris*	Fabaceae	Fruits	Methanol	HPLC–ESI/MS/MS, NMR	[80]
	*Tussilago farfara* L	Asteraceae	Flowers	Methanol	LC/MS/MS, TLC.NMR	[81]
	*Hypericum afrum Lam*	Hypericaceae	Aerial part	Chloroform, ethyl acetate and n-butanol	HR-ESI–MS, NMR	[55]
	*Guiera senegalensis*	Combretaceae	Leaves	Ethanol	HPTLC, NMR,	[56]
	*Cratoxylum formosum*	Hypericaceae	Leaves	Methanol	CC,1D- 2D-NMR, ESI-MS	[82]

CE (Capillary electrophoresis); HPLC (High performance liquid chromatography); RP-HPLC (Reversed-phase HPLC); QTOF MS (Quadrupole time of tlight tass spectrometer); RTCC (Reversed phase column chromatography); EI-MS-(electron ionization mass spectroscopy); LC/MS/MS (Liquid chromatography mass spectroscopy); HSQC (Heteronuclear single quantum coherence spectroscopy); HMBC (Heteronuclear Multiple Bond Correlation spectroscopy); HPLC-TOF/MS (High-performance liquid chromatography-time-of-flight mass spectrometry); RSM (Response surface methodology); RTPCR (Reverse transcription polymerase chain reaction); PVPP (polyvinylpolypyrrolidine column); HR-ESI–MS (high-resolution electrospray ionization mass spectrometry); ESI-Q-TOF-MS (electrospray ionization with quadrupole time-of-flight mass spectrometry); HPLC-Q-TOF-MS (high-performance liquid chromatography coupled with quadrupole time of flight mass spectrometry); EI-MS (electron impact mass spectrometry); UPLC-MS/MS (Ultraperformance liquid chromatography mass spectroscopy); HR-ESI–MS (high-resolution electrospray ionization mass spectrometry).

## 2 Material and methods

*Articles search* strategy: Using the databases from PubMed, Google Scholar, ScienceDirect, Scopus, ClinicalTrials.gov, and Wiley Online Library, a literature search was done to compile the findings about the shared biological activities of five significant flavonoids. ‘Flavonoids’, ‘apigenin’, ‘galangin’, ‘hesperetin’, ‘kaempferol’, ‘quercetin’, ‘myricetin’, ‘naringenin’, ‘antidiabetic’, ‘anti-inflammatory’, ‘antimicrobial’, ‘antioxidants’, ‘antiviral’, ‘cytotoxic’, ‘lipid-lowering’, ‘isolated from’ etc. were among the terms used in the searches. Only scientific journals that have undergone peer review were taken into account. After meticulously screening titles and abstracts of all identified publications to ensure they met the inclusion criteria, we included more than two hundred articles which satisfied these criteria. Flavonoids extracted from dietary sources and exhibiting antidiabetic, anti-inflammatory, antimicrobial, antioxidant, antiviral, cytotoxic, and lipid-lowering activities are considered for this review. All of these substances were chosen because they all fall under the category of most common dietary flavones and exhibited all of the aforementioned activities. Moreover, they can be found in numerous popular food sources like fruits and vegetables.

## 3 Isolation of concerned flavonoids

These bioactive flavonoids were extracted from different medicinal plants using different chromatographic separation procedures and they were identified using the various spectroscopic methods listed in Table 1.

## 4 Biological activities

### 4.1 Apigenin

Apigenin, or 4′, 5, 7-trihydroxy flavone, is a kind of flavonoid presented in many temperate and tropical vegetables and fruits, including celery, onion, orange, chamomile, and malt [83,84]. Certain plant-based beverages, such as tea and sauces, also contain apigenin [85]. It is a class of flavone, usually yellow crystalline powder that serves as an aglycone for a number of glycosides that are present in nature. It is water-insoluble but soluble in organic solvents [15]. This substance has long been employed as an anti-inflammatory and antioxidant agent [83].

#### 4.1.1 Antidiabetic activities

Apigenin as well as its two C-glycosylated derivatives namely vitexin and isovitexin manifested antidiabetic activity by inhibiting rat lens aldose reductase (RLAR), human recombinant aldose reductase (HRAR), advanced glycation endproducts (AGE), and protein tyrosine phosphatase 1B (PTP1B) formation. Here Apigenin showed IC_50_ values against RLAR, HRAR, AGE, and PTP1B were 0.97 ± 0.26, 11.65 ± 0.07, 204.14 ± 9.31, and 24.76 ± 2.17 μM respectively, however, these values for vitexin and isovitexin were 1.47 ± 0.08, 12.07 ± 0.03, 243.54 ± 8.86, and 7.62 ± 0.21 μM; and 0.49 ± 0.08, 0.13 ± 0.03, 175.66 ± 3.73, and 17.76 ± 0.53 μM respectively [86].

When administered in 25 and 50 mg doses, apigenin showed an antidiabetic effect in rats with adrenaline-induced hyperglycemia. The blood glucose levels peaked after 30 min were found at 65.73% and 52.88%, respectively for 25 and 50 mg doses of the compound, versus the untreated control (97.86%) and glibenclamide (63.90%) [87]. However, an *in silico* study illustrated that the compound exhibited the strongest binding affinity against glucose transporter type 3 (GLUT-3) receptor indicating its potential as an antidiabetic agent by modulating the GLUT-3 related pathways [88].

#### 4.1.2 Anti-inflammatory activities

The anti-inflammatory activity was exhibited by apigenin in mice with carrageenan-induced acute paw oedema, where both the doses of 25 and 50 mg/kg markedly reduced paw oedema. Also, the compound greatly decreased tumor necrosis factor's (TNF-α) ability to active nuclear factor kappa B (NF-κB) and transactivation of NF-κB, which acts as a crucial mediator of inflammatory reactions and controls various elements of the immune system's innate as well as adaptive functions [89,90].

Another research manifested that apigenin conveyed anti-inflammatory activity in pylori-infected MKN45 cells, where treatments with apigenin (9.3–74 μM) significantly raised the NF- -κB inhibitor alpha (IκBα) expression, blocked the activation of NF-κB, and reduced the production of inflammatory factors including cyclooxygenase-2 (COX-2), intercellular adhesion molecule 1, reactive oxygen species, interleukin-6 (IL-6), and interleukin-8 (IL-8) [91].

#### 4.1.3 Antimicrobial activities

The compound has shown antibacterial activity against *Pseudomonas aeruginosa, Salmonella typhimurium, Proteus mirabilis, Klebsiella pneumoniae,* and *Enterobacter aerogenes*, the most prominent activity has been shown against *S.typhimurium* and *P. mirabilis* with a zone of inhibition of 17.36 ± 0.18, and 19.12 ± 0.01 mm respectively, also the zone of inhibition were 12.24 ± 0.41, 10.52 ± 0.38, and 14.02 ± 0.03 mm against *P. aeruginosa, K. pneumoniae* and *E. aerogenes* [16]. Apigenin's antibacterial activity involves targeting nucleic acid processing enzymes, cell wall/membrane synthesis, and potential interactions with RNA polymerase and gyrase/topoisomerase IV, as indicated by its clustering with antibiotics like rifampicin and norfloxacin. Additionally, apigenin affects the d-Alanine: d-Alanine ligase and type II fatty acid synthetic pathway involved in cell membrane/wall synthesis [83].

Apigenin exhibited antifungal actions against the fungus *Candida albicans*, where it increased mitochondrial calcium uptake of the fungus in a manner dependent on dose, which was followed by mitochondrial dysfunction, membrane potential loss, and elevated mitochondrial mass and reactive oxygen species. Moreover, it led to an intracellular redox imbalance as shown by an uptick in ROS, glutathione oxidation, and lipid peroxidation. In the meantime, a dose of 2.5 μg/mLof the compound notably reduced the viability of *C. albicans* cells which was half the concentration of the previously described minimum inhibitory concentration of 5 μg/mL [18].

#### 4.1.4 Antioxidant activities

One experiment showed that in low doses (10, 20, and 40 mg/kg b.w.) apigenin shields rat livers from oxidative damage brought on by reactive oxygen species via lowering lipid peroxidation and membrane protein deterioration along with the secretion of blood serum enzyme indicator like, ALP, alaaminotransferase (ALT), aspartate transaminase (AST), and lactate dehydrogenase (LDH) [92].

Another experiment demonstrated that 14 days of administration of 25 mg/kg bw of apigenin resulted in protective activity against N-nitroso diethylamine-induced and phenobarbital-promoted oxidative stress in carcinogenic rats. Increased levels of enzymatic and non-enzymatic antioxidants like superoxide dismutase, catalase, glutathione peroxidase, reduced-glutathione, vitamin C, and vitamin E both in the liver and kidney were observed by this treatment, however, the compound greatly lowered lipid peroxidation levels in both liver and kidney [93].

#### 4.1.5 Antiviral activities

Through inhibition of the synthesis of viral DNA, mRNA, and protein of the buffalopox virus, Apigenin exhibited antiviral activity. The EC_50_ of apigenin was found to be 51.70 ± 3.40 ng/mL, and it strongly inhibited viral replication in a manner of dose [94]. This antiviral action against the buffalopox virus involves the host-directed mechanism of targeting cellular ERK/MNK1/eIF4E signaling, as observed by reduced eIF4E phosphorylation during virus replication [94].

By preventing the synthesis of certain proteins and viral factories, apigenin has been demonstrated to be effective against the African swine fever virus. There were 44%, 60%, and 74% fewer viral factories after exposure to doses of 12.5, 25, and 50 μM, respectively [95].

#### 4.1.6 Cytotoxic activities

Research showed that apigenin markedly increases the cytotoxic activity of cisplatin. In combination with cisplatin, apigenin decreased cell proliferation, aided mitogen-activated protein kinase activation (MAPK) and subsequent phosphorylation of p53, which resulted in p53 accumulation and an increase in proapoptotic proteins [96].

It has been demonstrated that apigenin can be administered orally in doses of 20 and 50 μg/mouse/day for 20 weeks considerably slowing the growth of prostate tumors and complete eradication of lymph node metastases from distant sites, the lungs, and the liver Moreover, the therapy increased E-cadherin levels while lowering nuclear β-catenin, c-Myc, and cyclin D1levels [97].

#### 4.1.7 Lipid-lowering activities

Apigenin exhibited an antihyperlipidemic effect on hyperlipidemic rats, where the compound decreased the levels of elevated total cholesterol (TC), triglyceride (TG), low density lipoprotein (LDL) concentration, and the protein expression of oxidized low-density lipoprotein receptor 1 (LOX-1), as well as elevated the lowered level of high density lipoprotein (HDL) concentration and the Bcl-2/Bax ratio [84].

Another research demonstrated that a 14-day treatment with apigenin at a dose of 50 mg/kg/day substantially lowered hyperlipidemia in high-fat diet-induced hyperlipidemic rats as evidenced by decreased TC, LDL, and TG levels and elevated HDL concentrations versus the positive control group. Where the concentrations found for cholesterol, HDL, LDL, very low density lipoprotein (VLDL), and TG were 74.90 ± 10.50, 123.30 ± 8.00, 10.20 ± 1.00, 12.10 ± 2.20, and 70.00 ± 2.00 mg/dL respectively by the compound and 286.00 ± 87.00, 35.30 ± 2.50, 31.70 ± 1.50, 45.00 ± 4.00, 197.60 ± 35.00 mg/dL respectively by the positive control group [98].

Additionally, in palmitic acid-induced human hepatoblastoma cell line (HepG2) cells, the substance increased autophagosome formation, stimulated autophagic flux in the cytoplasm, decreased the content of lipids, and co-localized lipid droplets with LC3, demonstrating that apigenin promoted autophagic lipid breakdown and triggered autophagy [99].

In summary, apigenin and its derivatives vitexin and isovitexin have powerful effects in treating diabetes by stopping certain enzymes that play a role in the disease. Apigenin's efficacy is highlighted by its low IC_50_ values against various targets. Apigenin has strong anti-inflammatory effects, reducing swelling and preventing a protein called NF-ƙB from becoming activated. Additionally, it has strong abilities to kill bacteria and fungus, and it also disrupts important functions in cells. Apigenin also has antioxidant qualities that protect tissues from oxidative damage and encourage the activity of antioxidants. Its ability to prevent viral DNA and protein production makes its antiviral potential evident. Moreover, apigenin has potential in treating cancer by helping cisplatin to better kill cancer cells and prevent tumors from growing and spreading. Finally, it can help lower fats in the blood, like triglycerides and cholesterol, and also helps break down fats through a process called autophagy. Apigenin is a substance that is being studied a lot in biomedical research because it has many different qualities that could possibly help with treating different illnesses.

### 4.2 Galangin

A flavonol called galangin (3,5,7-trihydroxy flavone) is found in the Asian folk remedy *Alpinis officinarum* and the propolis that honeybees naturally generate [100]. It has a molecular formula of C_15_H_10_O_5_ and a molecular weight of 270.24 [101]. Two benzene rings connected by a C-3 aliphatic chain; a 2,3-double bond with a 3-hydroxyl group in the C-ring, and a heterocyclic pyran ring without a hydroxyl group in the B-ring; five and seven dihydroxyl groups in the A-ring, and a 2,3-double bond with a 3-hydroxyl group are the structural components of galangin [102]. It possesses several medicinal activities including anti-inflammatory, antiviral, anticancer, antioxidant, and anti-obesity effects [103].

#### 4.2.1 Antidiabetic activities

Galangin manifested hyperglycemic activity in streptozotocin-induced hyperglycaemic rats, where the dose of 4, 8, and 16 mg/kg b.w significantly decreased glucose levels with values of 79.86 ± 0.80, 117.10 ± 11.32, and 140.53 ± 7.24 mg/dL respectively compared to diabetic control 281.10 ± 9.57 mg/dL. In addition, the treatment also increased insulin levels with values of 8.14 ± 0.79, 12.08 ± 1.04, and 10.45 ± 1.72 μU/mL for 4, 8, and 16 mg/kg b.w dose compared to diabetic control 6.01 ± 0.44 μU/mL [104]. A study revealed that galanin's antidiabetic activity is linked to its interaction with the Dipeptidyl peptidase-4 (DPP-4) pathway. The compound binds to the DPP-4 receptor, exerting an antagonistic effect that results in a reduction of glucose levels [105].

Research demonstrated antidiabetic activity of galangin in streptozotocin-induced diabetic rats, where the dose of 4, 8, and 16 mg/kg/day of the substance tested against these rats for 45 congestive days, and 8 mg/kg/day has manifested the most prominent activity compared to standard glibenclamide by reducing blood glucose level. However, treatment with 8 mg/kg/day galangin for 45 days notably increased the levels of some membrane-bound enzymes in erythrocytes and tissues, including total ATPases, sodium-potassium-ATPase, calcium-ATPase, and magnesium-ATPase, which were altered in diabetic conditions [106].

#### 4.2.2 Anti-inflammatory activities

Galangin exhibited antiinflammatory activity on RAW 264.7 murine macrophage cells. where the drug significantly decreased the amounts of inducible nitric oxide synthase (iNOS) protein expression in activated macrophages as well as nitric oxide (NO) generation brought on by lipopolysaccharide (LPS) stimulation. Yet, the substance dose-dependently decreased mRNA levels of proinflammatory genes like iNOS and cytokines like interleukin-1b (IL-1b) and IL-6 in LPS activated macrophages. Galangin also prevented LPS-activated macrophages from producing IL-1b. These findings suggest that galangin reduces inflammation in LPS-activated macrophages by inhibiting extracellular signal-regulated kinase (ERK), NF-kB-p65, and pro-inflammatory gene expression [100].

Galangin was demonstrated to have anti-inflammatory effect in a different investigation using LPS-stimulated RAW264.7 macrophages, where the compound significantly decreased the production of NO, inducible NO synthase, IL-6, the NF-κB and suppressed the LPS-induced activation of ERK-1/2 and c-Jun N-terminal kinase [107].

#### 4.2.3 Antimicrobial activities

Minimum inhibition concentration was observed by galangin against vancomycin-intermediate *Staphylococcus aureus* standard strain Mu50, methicillin-susceptible *S. aureus* standard strain ATCC25293 and methicillin-resistant *S. aureus* standard strain N315 was 32 μg/mL, indicating antibacterial activity of the compound against *S. aureus*. Additionally, doses of 4, 8, and 16 μg/mLof the compound prominently blocked *S. aureus* growth [108].

Another study has proven that galangin exhibited bacteriocidal activity against methicillin-resistant *S. aureus*, *Enterococcus* spp., and *P. aeruginosa* with the zone of inhibition of 10.90 ± 1.40, 9.80 ± 1.00, and 6.40 ± 4.60 mm respectively [109].

#### 4.2.4 Antioxidant activities

A study was investigated the antioxidant activity of galangin using the trolox equivalent antioxidant capacity (TEAC) assay, where he observed antioxidant activities of the compound at concentrations ranging from 1 to 2000 μM, but the active trolox concentration was 10 μM [110].

Furthermore, the compound demonstrated antioxidant activity against damaged mitochondria in the livers of streptozotocin (STZ) induced diabetic rats. In this study, a dose of 8 mg/kg BW galangin considerably lowered oxidant namely thiobarbituric acid reactive substance levels while boosting enzymatic antioxidants including superoxide dismutase, glutathione peroxidase, and non-enzymatic antioxidants including reduced glutathione levels [111].

#### 4.2.5 Antiviral activities

Galangin displayed potent *in vitro* antiviral activity against herpes simplex virus type 1 (HSV-1), as evidenced by its EC_50_ values of 2.5 μM and a high selective index of 400 (calculated as the ratio of 50% cytotoxic effective concentration to 50% effective concentration). At a concentration of 100 μM, it exhibited an impressive 81% inhibition of the virus, and even at a lower concentration of 25 μM, a substantial 45% inhibition was still observed [112].

In a recent *in silico* study, ganalngin demonstrated highly encouraging antiviral potential against COVID-19. The compound displayed a very favorable binding affinity with the spike glycoprotein of severe acute respiratory syndrome coronavirus (SARS-CoV-2), scoring −8.2 kcal/mol, surpassing dexamethasone's score of −7.9 kcal/mol [113].

#### 4.2.6 Cytotoxic activities

The natural compound galangin inhibited ovarian cancer cells A2780/CP70 and OVCAR-3 more effectively than normal cells I0SE364, with IC_50_ values of 42.3, 34.5, and 131.3 μM, respectively. DR5 proteins are upregulated by cleaved caspase-3, which is likewise elevated by galangin. The suppression of phosphorylated AKT and p70S6K also shows that the AKT/p70s6K pathway is involved in apoptosis [114].

Another research demonstrated that galangin strongly inhibits TPA(2-O-tetradecanoylphorbol-13-acetate)-induced protein expressions of protein kinase Cα (PKCα), resulting in the phosphorylation of ERK1/2. This phosphorylation leads to a decrease in the activity and expression of matrix metalloproteinase-2 (MMP-2) and matrix metalloproteinase-9 (MMP-9). Furthermore, GA inhibits the phosphorylation of inhibitor of IκBα, as well as the activity of NF-κB and its binding to activator protein 1 (AP-1). This overall inhibition effectively suppresses the activity of MMP-2 or MMP-9 and contributes to the inhibition of hepatocellular carcinoma metastasis [115].

Galangin inhibited the human cervical cancer cell line (HeLa) cells at an IC_50_ value of 50 μM, which resulted in enhancing the intracellular ROS levels in HeLa cells, leading to DNA damage, and triggering cell death. Furthermore, the compound decreased the activity of Nrf-2, a transcription factor involved in redox signalling, as well as glyoxalase-1, an enzyme involved in the detoxification of the deadly metabolite methylglyoxal [116].

#### 4.2.7 Lipid-lowering activities

Antihyperlipidemic potency was observed by galangin in streptozotocin (STZ)-induced hyperglycaemic rats, the substance prominently reduced elevated TC, TG, LDL, VLDL, free fatty acid (FFA), and phospholipid levels due to diabetics, as well as increased the reduced HDL level due to the disease condition [104].

A study inspected the hypolipidemic action of galangin and its two metabolites including galanin-3-O-β-d-glucuronic acid and galanin-7-O-β-d-glucuronic acid against female Sprague Dawley rats and observed that the compound and both of its metabolites were found to have potential hypolipidemic potency by down-regulating lipogenic genes like SREBP-1a, SREBP-1c, and SREBP-2 transcription factors, as well as suppressed genes like FAS, ACC, and HMGR [117].

To put it simply, Galangin shows promising effects for reducing diabetes in rats by lowering their blood sugar levels and increasing the amount of insulin in their bodies. The way it works with DPP-4 helps it reduce diabetes. Galangin also exhibits potent anti-inflammatory activities, inhibiting iNOS protein expression and proinflammatory gene expression in activated macrophages. Additionally, it displays strong antimicrobial properties against *Staphylococcus aureus* and methicillin-resistant strains. Galangin has strong antioxidant properties, which protects against damage from harmful substances in diabetic rats. Moreover, it demonstrates a positive affinity towards binding with SARS-CoV-2. In terms of cytotoxicity, it inhibits various cancer cell lines, including ovarian cancer and cervical cancer cells, through multiple pathways. Furthermore, galangin has notable lipid-lowering effects, reducing elevated lipid levels and down-regulating lipogenic genes. These diverse and potent biological activities highlight galangin's potential as a therapeutic agent in diabetes, inflammation, infections, cancer, and cardiovascular disorders, making it a promising candidate for further research and development in the field of medicine.

### 4.3 Hesperetin

Hesperetin, the aglycone of hesperidin (the main flavone glycoside present in citrus fruits) also recognized as 3′, 5, 7-trihydroxy-4′-methoxy flavanone, is dihydro flavone with the chemical formula C_16_H_14_O_6_. It is a member of the group of flavonoids known as flavanones and one of the most common flavonoids found in citrus fruits, with a variety of pharmacological effects [118,119]. However, it has a variety of activities, including anti-inflammatory, antibacterial, anticarcinogenic, and antioxidant actions [120].

#### 4.3.1 Antidiabetic activities

Hesperetin manifested notable antidiabetic activity against STZ-induced diabetic rats, where it decreased glucose level with a value of 129.60 ± 4.16 mg/dL compared to diabetic control 8284.80 ± 2.39 mg/dL, however, increased insulin and glucagon level with values of 0.94 ± 0.02 mIU/L and 75.29 ± 1.61 Pg/mL respectively, compared to diabetic control 0.45 ± 0.02 mIU/L and 66.98 ± 1.20 Pg/mL respectively. However, treatment of the compound improved the level of glucose transporter 2 and glucose transporter 4 and the activities of glucokinase and hexokinase in the rat's pancreatic tissues in comparison to diabetic control, as well as comparably decreased the levels of pancreatic glucose-6-phosphate and fructose-1,6-bisphosphate, 6-phospho gluconate dehydrogenase and glucose-6-phosphate dehydrogenase enzymes. Additionally, this therapy altered the levels of the expression of the insulin receptor, phosphoinositide 3-kinase, AMP-activated protein kinase, caspase-3, and interleukin-1 (IL-1) [33].

Similarly, treatment with 40 mg/kg b.w hesperetin for 45 days on STZ-induced diabetic rats showed potential antidiabetic activity, where the treatment suggestively abridged serum glucose level along with enhanced plasma insulin level. Also, the treatment notably reduced the activity of glycolytic enzymes namely glucokinase and glucose-6-phosphate dehydrogenase with values of 0.37 ± 0.03 u/h/mg protein and 3.31 ± 0.25 lU/l respectively compared to diabetic control 0.21 ± 0.02u/h/mg protein and 1.99 ± 0.15 lU/l respectively; gluconeogenic enzymes namely glucose-6-phosphatase and fructose 1,6 bis phosphatase 0.220 ± 0.03 and 0.420 ± 0.02 μmoles of Pi liberated/min/mg of protein respectively related to diabetic control 0.290 ± 0.05 and 0.670 ± 0.03 μmoles of Pi liberated/min/mg respectively; and hepatic glycogen level with a value of 17.12 ± 1.31 mg/g liver compared to diabetic control 10.75 ± 0.82 mg/g liver [119].

#### 4.3.2 Anti-inflammatory activities

In a study anti-inflammatory activity of hesperetin was tested against LPS-induced neuroinflammation-affected mice, where administration of hesperetin prominently reduced the expression of inflammatory mediators namely phosphorylated NF-κB, TNF-α, and IL-1β, as well as downgraded the expression of inflammatory cytokines by ameliorating Toll-like receptor-4-mediated ionized calcium-binding adapter molecule 1 (Iba-1)/glial fibrillary acidic protein (Iba-1/GFAP) expression [121].

15 days of treatment with hesperetin in partial sciatic nerve ligation-affected rats showed potential antiinflammatory activity by reducing inflammatory markers including IL-1β and IL-6, herein dose of 20 mg/kg demonstrated values of 890.70 ± 60.50 and 2713.00 ± 84.50 pg/mL respectively, the values for 50 mg/kg were 787.60 ± 58.70 and 2410.00 ± 84.90 pg/mL respectively, in the comparison control group showed values of 2580.00 ± 171.80 and 3316.00 ± 81.50 pg/mL respectively. Additionally, the treatment significantly reduced another anti-inflammatory marker, TNF-α paralleled to the control group through a manner of dose. Moreover, this treatment improves some behavioural parameters including paw withdrawal latency in radiant heat hyperalgesia, paw withdrawal latency in cold allodynia, paw withdrawal threshold in static mechanical hyperalgesia, paw withdrawal threshold in mechano-tactile, and paw withdrawal latency in tactile mechanical [122].

#### 4.3.3 Antimicrobial activities

Hesperetin demonstrated moderate antifungal activity against *C. albicans*, *Candida tropicalis*, and *Malassezia furfur*, where the compound was found active against *C. tropicalis* at concentrations of 15, 20, and 25 mg/mL; for *M. furfur*, activity was found at concentrations of 20, 25 mg/mL, and only the concentration of 25 mg/mL was found active against *C. albicans* [123].

Another research demonstrated the antimicrobial activity of hesperetin where the compound showed antifungal activity against some *Candida* fungal strains including *C. albicans* 10/15, *C. albicans* 10/15, *C. albicans* 475/15, *C. albicans* ATCC 10231, *C. parapsilosis* ATCC 22019, *C. tropicalis* ATCC 750, C. krusei H1/16, *C. glabrata* 4/6/15 with a minimum inhibitory concentration (MIC) of 0.165 mg/mL. Also, the compound exhibited antibacterial activity against *S. aureus* ATCC 11632 with MIC of 4 mg/mL [124].

#### 4.3.4 Antioxidant activities

Hesperetin demonstrated antioxidants activity in streptozotocin-induced diabetic Wistar albino rats, herein the compound altered antioxidants marker levels like reduced glutathione (GSH), superoxide dismutase (SOD), catalase (CAT), glutathione peroxidase (GPx), thiobarbituric acid reactive substances (TBARS), and AGE compared to diabetic control, where the compound improved the level of GSH, SOD, CAT, and GPX with values of 49.80 ± 0.63 nmol/g tissue, 8.00 ± 0.30, 27.90 ± 0.30, and 5.50 ± 0.04 U/g tissue respectively compared to diabetic control 41.30 ± 0.72 nmol/g tissue, 2.10 ± 0.05, 17.00 ± 0.19, and 3.20 ± 0.11 U/g tissue respectively. Also, treatment with the compound comparably decreased TBARS and AGE levels with values of 12.80 ± 0.36 nmol/g tissue and 97.40 ± 0.54 ng/g tissue respectively compared to diabetic control 17.30 ± 0.41 nmol/g tissue and 103.60 ± 0.66 respectively [33].

Another study showed that hesperetin exhibited antioxidant activity in STZ-induced diabetic rats, where 45 days of treatment with a dose of 40 mg/kg b.w of the constituent significantly reduced plasma SOD, pancreas SOD, plasma CAT, pancreas CAT, plasma GST, pancreas GST, plasma GPx, and pancreas GPx with values of 7.50 ± 0.57, 3.96 ± 0.30, 57.57 ± 4.41, 5.24 ± 0.40, 26.65 ± 2.05, 2.76 ± 0.21, 32.99 ± 2.52, and 0.35 ± 0.03 U/mg protein compared to diabetic control 4.91 ± 0.37, 2.38 ± 0.17, 42.01 ± 3.20, 2.70 ± 0.21, 16.87 ± 1.28, 1.37 ± 0.10, 21.00 ± 1.02, and 0.18 ± 0.01 U/mg protein respectively [119].

In adult rats exposed to lead, the antioxidant effects of hesperetin at a dose of 50 mg/kg/day were examined. where the treatment altered the serum biochemical parameters, including amplified lipid peroxide and diminished GSH, SOD, CAT, and GPx levels in liver and kidney tissues into normal states, suggesting that hesperetin may be crucial in the treatment of lead-induced neurotoxicity by limiting the negative effects of lead by lowering oxidative damage [125].

#### 4.3.5 Antiviral activities

By lowering the effectiveness of chikungunya virus replication and down-regulating the synthesis of viral proteins involved in replication, hesperetin demonstrated antiviral action against Chikungunya virus intracellular replication. However, the substance's IC_50_ value for blocking chikungunya virus replication activity was 8.500 μM as well as the selective index was 23.34 [126].

In a pathogen challenge study, hesperetin was administered to crayfish (*Procambarus clarkii*) infected with white spot syndrome virus (WSSV). The results showed that hesperetin treatment effectively lowered mortality rates caused by WSSV, while also reducing the viral load (VP28 copies). Moreover, hesperetin increased the expression of innate immune-related genes (NF-ƙB and C-type lectin) and positively impacted critical immune parameters like total hemocyte count, phenoloxidase, and superoxide dismutase activity. However, it was noteworthy that hesperetin also led to a significant increase in hemocyte apoptosis rates in crayfish, regardless of WSSV infection status [127].

#### 4.3.6 Cytotoxic activities

Hesperetin activated the PI3KAkt signalling pathway in antigen-presenting cells (APCs), improved cytotoxic T lymphocyte (CTL) reactions, and neutralized tolerogenic T cells in a study of hesperetin with incapacitated B16F10 melanoma cells. However, when combined with inactivated B16F10 cells, the compound inhibited tumour growth in mice, resulting in enhanced complete existence when linked to the effect of the deactivated B16F10 cell vaccine [118].

In another research, hesperetin exhibited cytotoxicity in 4T1 murine metastatic breast cancer cells, and its combination with the chemotherapeutic agent doxorubicin demonstrated a synergistic effect by increasing G2/M phase cell cycle arrest and apoptosis induction. Also in 4T1 cells, the combination of Hst and Dox inhibited migration and decreased MMP-9 expression [128].

In A431 human skin carcinoma cells, hesperetin was tested for cytotoxic and pro-apoptotic activities. Herein, the substance-induced apoptosis in A431 cells by altering the expression levels of ERK, JNK (c-Jun NH2-terminal Kinase), and p38. This was accomplished by stimulating changes in the MAPK signalling pathway. The levels of cyclin A2, B1, D1, D3, and E1 were controlled by hesperetin. Furthermore, p21, Bcl-2, and Bax were among the proteins in the apoptotic pathway whose levels were altered [129].

#### 4.3.7 Lipid-lowering activities

Research demonstrated the hypolipidemic potency of hesperetin by altering the lipid parameters namely TC, TG, HDL, and LDL in high-fat diet-fed and STZ-induced diabetic rats. A 4-week treatment with 40 mg/kg BW/day of the compound notably reduced TC, TG, and LDL levels with values of 101.10 ± 1.67, 119.00 ± 1.58, 80.50 ± 1.58 mg/dL respectively associated with the diabetic control group 177.0 ± 1.58, 146.0 ± 1.58, 136.9 ± 1.60 mg/dL respectively. Moreover, the treatment increased HDL level with a value of 19.0 ± 0.88 mg/dL compared to diabetic control 13.2 ± 0.66 mg/dL [33].

Another research demonstrated, 45 days of management with 40 mg/kg b.w hesperetin on STZ-induced diabetic rats improved lipid profile by reducing TC, TG, FFA, and phospholipids levels with values of 5.01 ± 0.41, 5.12 ± 0.39, 17.61 ± 1.29, and 25.01 ± 1.91 respectively, where the values for diabetic control group were 6.80 ± 0.52, 8.23 ± 0.63, 23.00 ± 1.75, and 31.11 ± 2.37 mg/g wet tissues respectively indicating prominent antihyperlipidemic activity [119].

In conclusion, hesperetin is a versatile medicine that has strong abilities to treat diabetes. It can effectively lower glucose levels, increase insulin and glucagon levels, and adjust important enzymes and receptors related to glucose metabolism. Moreover, its anti-inflammatory properties are evident in mitigating neuroinflammation and peripheral inflammatory markers, offering potential in inflammatory conditions and pain management. Hesperetin can help fight infections because it is effective against different types of harmful bacteria. Its powerful antioxidant properties help protect cells from oxidative stress, which could be helpful for disorders related to oxidative stress. Furthermore, hesperetin displays promising antiviral activities, particularly against Chikungunya virus and white spot syndrome virus, offering avenues for antiviral research. This discovery opens the door for further research on antiviral treatments. Additionally, it can kill cancer cells and make chemotherapy medications work better, which shows that it could be useful in cancer treatment. Lastly, hesperetin's ability to regulate lipid profiles suggests its utility in managing hyperlipidemia. In summary, hesperetin's diverse pharmacological activities underscore its significance in various therapeutic applications, making it a valuable subject for further research and development.

### 4.4 Kaempferol

Kaempferol also known as 3,5,7-trihydroxy−2-(4-hydroxy phenyl) −4H−1-benzopyran−4-one (Molecular weight-286.2 g/mol is a naturally occurring yellow colour flavonol that is mostly formed from the rhizome of the ginger family and is found in a variety of plant species, including tea, broccoli, purple cabbage, beans, chicory, leeks, tomatoes, strawberries, and grapes [130,131]. It is a tetrahydroxy flavone, and the hydroxy groups are in 3, 5, 7, and 4′ positions [132]. Cardioprotective, neuroprotective, anti-inflammatory, antidiabetic, antioxidant, antibacterial, and anti-cancer effects have been to linked with kaempferol and its glycosylated derivatives [133].

#### 4.4.1 Antidiabetic activities

When given orally for 12 weeks to STZ-induced diabetic mice, kaempferol (50 mg/kg/day) considerably condensed the occurrence of explicit diabetes from 100% to 77.8% and significantly improved hyperglycemia. This result was accompanied by an upsurge in glucose oxidation of diabetic mice's muscles and a decrease in the synthesis of hepatic glucose. However, the therapy consistently restored hexokinase activity while suppressing hepatic pyruvate carboxylase activity and gluconeogenesis [134].

In high-fat-fed rats, 92 days of therapy with 0.15% dietary kaempferol showed good anti-diabetic action by lowering serum HbA1c (hemoglobin A1c 35) levels and improving insulin resistance [135].

#### 4.4.2 Antiinflammatory activities

By dramatically reducing the levels of monocyte-derived chemokine (MDC), interferon gamma-induced protein 10 (IP-10), and IL-8 produced by LPS in THP-1 cells, kaempferol demonstrated anti-inflammatory efficacy. However, the compound inhibited the phosphorylation of upstream c-raf and MEK1/2 as well as the LPS-induced MAPK and growth-related oncogene-α (GRO-α) pathways [136].

Kaempferol reduced the production of matrix metalloproteinases-1 (MMP-1), matrix metalloproteinases-3(MMP-3), COX-2, and prostaglandin E2 (PGE2) in response to interleukin-1β (IL-1β) as well as the proliferation of both unstimulated and IL-1β-stimulated rheumatoid arthritis synovial fibroblasts. Additionally, kaempferol prevented ERK1/2, p38, JNK, and NF-κB activation brought on by IL-1β. These findings suggest that kaempferol reduces synovial fibroblast proliferation as well as the synthesis of MMPs, COX-2, and PGE2, which are associated with articular inflammation and degeneration in rheumatoid arthritis [137].

According to a study, the inflammatory cascade that was triggered by the management of human umbilical vein endothelial cells with a cytokine combination increased the expression of vascular cell adhesion molecule-1, intercellular adhesion molecule-1, endothelial cell selection, and other inflammatory mediators like inducible NO synthase, cyclo-oxygenase-2, NF-κB, and activator protein-1 (AP-1), whereas treatments with kaempferol greatly decreased their expression [138].

#### 4.4.3 Antimicrobial activities

Kaempferol effectively suppressed *Helicobacter pylori* growth in a concentration-dependent manner in the adenocarcinoma gastric cell line, exhibiting an *in vitro* antibacterial activity [139].

Kaempferol demonstrated significant antimicrobial activity against the planktonic and biofilm forms of *Candida parapsilosis* complex, reducing the metabolic activity and biomass of growing biofilms. It also caused an overall reduction in biofilm biomass in mature biofilms, indicating its potential as a sustainable resource for controlling fungal biofilms [140].

Also, the antifungal activity of kaempferol was observed in a human colon cancer cell line named HT-29 against *Candida albicans* with an IC_50_ value of 25 μg/mL and 15.00 ± 2.00 mm zone of inhibition [141].

#### 4.4.4 Antioxidant activities

Kaempferol demonstrated antioxidant activity using a variety of reactive oxygen species (ROS) scavenging techniques. The compound demonstrated notable scavenging activity against hypochlorous acid (HOCl) with an IC_50_ value of 0.0020 mM, against chloramines with an IC_50_ value of 0.092 mM, and against superoxide (O_2_^•-^) with an IC_50_ value of 0.243 mM. However, in nitric oxide (NO) scavenging assays, the compound inhibited a 21% concentration of NO with an IC_50_ value of 0.172 mM [142].

To find out the antioxidant kaempferol and its three glycosides including kaempferol-7-O-glucoside, kaempferol-3-O-rhamnoside and kaempferol-3-O-rutinoside were tested on LPS-induced RAW 264.7 cells, where kaempferol-7-O-glucoside showed good activity. However, by preventing concanavalin A (Con A)-induced activation of T cell proliferation and NO or ROS formation in LPS-induced RAW 264.7 macrophage cells, kaempferol demonstrated the most notable antioxidant effect [143].

#### 4.4.5 Antiviral activities

One of the most significant infections that threaten the world's pig industry is the *Pseudorabies* virus, a member of the Alphaherpesvirus family. Kaempferol showed antiviral action in mice against this virus. In comparison to acyclovir (Positive control), which had a survival rate of 16.67% after six days of infection, the compound treatment enhanced the survival rate by 22.22% over six days. In contrast, after six days' survival was zero per cent in the infected-untreated group. In particular, kaempferol was able to prevent the spread of viruses in the brain, lung, kidney, heart, and spleen, particularly the viral gene copies in the brain, which were reduced by over 700-fold. Additionally, Kaempferol drastically reduced the transcriptional levels of the early genes, such as EPO and TK, by inhibiting the transcription of the single immediate-early gene in the brain, IE180. Since kaempferol also suppressed the manifestation of the latency-associated transcript (LAT) in the brain, it was possible that it could prevent the virus latency. At 3 days post-injection, kaempferol administration might increase the serum levels of IL-1, IL-4, IL-6, TNF-α and IFN-γ which thereafter decreased to normal levels at 5 days post-injection [144].

In tests on the baby hamster kidney cell line, kaempferol was found to significantly suppress the Japanese encephalitis virus (JEV) infection, but significantly increase the infection of the dengue virus serotype 2 (DENV 2). In contrast, in a human embryonic kidney cell line, kaempferol did not exhibit any discernible antiviral effect against either virus [145].

It has been established that the SARS-CoV protein encoded by open-reading-frame 3a forms a cation-selective channel that may express itself in the infected cell. The channel's activity plays a role in the virus's release mechanism. Therefore, medications that block the ion channel can prevent the release of viruses. Therefore, the ability of kaempferol, kaempferol glycosides, and acylated kaempferol glucoside derivatives to inhibit the 3a channel was investigated for antiviral activity. The kaempferol glycoside juglanin, with an IC_50_ value of 2.30 μM, was the most efficient in inhibiting the 3a-mediated current. Additionally, kaempferol derivatives containing rhamnose residue appear to be extremely effective, indicating that kaempferol glycosides are promising candidates for coronaviruses' 3a channel proteins [146].

#### 4.4.6 Cytotoxic activities

On the human hepatoma cell line HepG2, the mouse colon cancer cell line CT26, and the mouse melanoma cell line B16F1, kaempferol demonstrated a substantial antiproliferation impact. In HepG2 cells, Kae also markedly reduced AKT phosphorylation and cleaved caspase-9, caspase-7, caspase-3, and PARP [21].

Also, the compound manifested antiproliferative activity by decreasing cell viability of HepG2 cells [147].

#### 4.4.7 Lipid-lowering activities

Kaempferol exhibited antihyperlipidemic activity in high-fat diet-fed hyperlipidemic mice, where various doses of the compound have shown dose-dependent antihyperlipidemic activity. Treatments lasting 8 weeks at doses of 75, 150, or 300 mg/kg/day decreased body weight gain, weights of the visceral fat pads, plasma cholesterol levels, coronary artery risk, and atherogenic indices. However, the most notable activity was seen at a dose of 300 mg/kg/day, which was approximately identical to the recommended dose of 100 mg/kg/day of fenofibrate. Additionally, the therapy alleviated hyperlipidemia and decreased visceral fat accumulation in experimental rats by upregulating hepatic peroxisome proliferator-activated receptor α (PPARα) expression, enhancing hepatic expression of acyl-CoA oxidase (ACO) and cytochrome P450 isoform 4A1 (CYP4A1), as well as increased lipid metabolism through the suppression of sterol regulatory element binding proteins (SREBPs) [148].

A research illustrated that, 92 days of treatment with 0.15% dietary kaempferol in high-fat-fed mice showed considerable antihyperlipidemic activity by lowering body weight, adipose tissue, serum TC, TG, LDL, NEFA, Leptin, and Leptin while raising serum HDL and both low- and high-molecular-weight adiponectin levels. Similarly, the therapy raised TC levels while decreasing TG levels in the liver. However, the treatment elevated the total lipid and TC count on faces, whereas TG and total bile acid count on faces decreased. Additionally, the therapy reduced SREBP-1c and PPAR--γ gene expression, indicating an improvement in the hyperlipidemic condition [135].

Another study showed the compound's hypolipidemic activity by enhancing LDL cholesterol clearance in human HepG2 cells through LDL uptake. Treatment with various concentrations of the compound, including 15, 30, and 45 M, resulted in 58.53, 46.72, and 40.77% LDL uptake, and it is clear that the compound's 15 M lowest concentration exhibited the most pronounced activity [147].

On the final note, kaempferol, administered orally, demonstrates a wide array of therapeutic effects, making it a promising candidate for diverse medical applications. In relation to diabetes, it greatly reduces the chances of getting diabetes and improves high blood sugar levels by increasing the breakdown of glucose and decreasing the production of glucose in the liver. It can help reduce inflammation in the body, which is helpful for conditions like arthritis. Kaempferol is effective against many bacteria and fungi, making it useful in fighting infections. It can fight against harmful substances in the body that cause damage, showing how it helps with problems related to stress on the body. Notably, kaempferol shows antiviral effects against significant viruses, including Pseudorabie*s* virus and Japanese encephalitis virus, offering prospects for antiviral research. Its ability to kill cancer cells shows that it could be used in cancer treatment, whether by itself or alongside other treatments. Furthermore, kaempferol has the ability to lower lipid levels and improve conditions of high cholesterol by controlling the amount of fat in the blood and helping to remove excess LDL cholesterol. Overall, these multifaceted pharmacological actions underline kaempferol's therapeutic potential across various health conditions, emphasizing its significance for further exploration and development in the field of medicine.

### 4.5 Myricetin

Myricetin, 3,5,7,3′,4′,5′-hexahydroxyflavone or 3,5,7-trihydroxy-2-(3,4,5-trihydroxyphenyl)-4-chromenone, a class of natural flavonoids with hydroxyl substitution at the 3, 5, 7, 3′, 4′, and 5’ positions are frequently distributed in fruits and vegetables [149,150]. In the form of glycosides, myricetin is present in berries, vegetables, herbs, and walnuts [151]. Myricetin is made up of crystals that are a light yellow colour and is soluble in polar solvents such as methanol, acetonitrile, and ethanol. The relative molecular mass of this substance is 318.24, and its chemical formula is C_15_H_10_O_8_ [150]. Several biological activities like antioxidant, pro-oxidant, anti-osteoporoti, anti-diabetic, anti-inflammatory, anti-Alzheimer, anti-cancer, hepatoprotective, cardioprotective, and gastroprotective activities were reported for the compound [152].

#### 4.5.1 Antidiabetic activities

Glucagon-like peptide 1 (GLP-1) is a receptor that regulates blood glucose levels by promoting insulin synthesis and secretion, inhibiting glucagon secretion, delaying stomach emptying, and promoting satiety. A dose of 250 mg/kg body weight, given twice daily for 40 days to laboratory mice, myricetin depicted glucoregulatory activity through agonizing the GLP-1 receptor, suggesting that the substance may have potential anti-type 2 diabetes mellitus effects [153].

In a separate *in vivo* study, myricetin was found to have anti-diabetic efficiency in laboratory rats. The substance repressed intestinal α-glucosidase and porcine α-amylase around 29 and 64% respectively with an IC_50_ value of 0.38 μM. Additionally, it inhibited 94% of yeast α -glucosidase, with an IC_50_ value of 5 μM [154].

#### 4.5.2 Anti-inflammatory activities

RAW 264.7 cells activated by LPS and a mouse model of LPS-induced lung injury were used to assess the anti-inflammatory effect of myricetin (*In vivo*). The *in vivo* test on the mouse model revealed that pretreatment with myricetin significantly reduced the severity of the histological changes, the activation of macrophages, and the onset of pulmonary oedema. The *in vitro* experiment on RAW 264.7 cells also showed that myricetin suppressed NF-κB p65 and AKT activation in the NF-κB pathway and JNK, *p*-ERK, and p38 activation in the MAPK signalling pathway, which are both involved in inflammatory responses [155].

In a different study, myricetin demonstrated anti-inflammatory effects against the *Porphyromonas gingivalis*-induced inflammatory response in host cells, where the dose of 62.5–125 g/mL myricetin suppressed the release of IL-6, IL-8, and MMP-3 by *P. gingivalis*-stimulated gingival fibroblasts and prevented NF-κB activation in a monocyte model [156].

#### 4.5.3 Antimicrobial activities

In a study, myricetin manifested an antimicrobial effect against some food-born strains of *Escherichia coli* and *Salmonella* (*S. paratyphi, S. cholerasuis* subsp., and *S. enteritidis*), where the compound inhibited all of the strains with MIC less than 20 ppm [157].

By compromising the integrity of the cell wall and greatly increasing membrane permeability, myricetin demonstrates strong antifungal action against *Candida albicans*. Myricetin's minimum inhibitory concentration (MIC) against *C. albicans* rises in the presence of sorbitol, showing the disruption of the cell wall that occurs. Propidium iodide staining of *C. albicans* cells under fluorescence microscopy is evidence of myricetin-induced early cell membrane disruption. The lipid composition and configuration of the cell membrane are impacted by enhanced membrane permeability in myricetin-treated *C. albicans* cells, as shown by crystal violet uptake and intracellular material leakage experiments [158].

#### 4.5.4 Antioxidant activities

Myricetin exhibits a strong antioxidant activity as shown by its capacity to scavenge intracellular reactive oxygen species (ROS), particularly 1,1-diphenyl-2-picrylhydrazyl (DPPH) radicals. As well, myricetin improves the activity and protein expression of several antioxidant enzymes, including glutathione peroxidase (GPx), catalase, and superoxide dismutase (SOD), which are impaired by oxidative stress brought on by hydrogen peroxide (H_2_O_2_). Notably, myricetin efficiently protects against cellular DNA and lipid damage by preventing DNA strand breaks and reducing membrane lipid peroxidation. It suggest that myricetin inhibits ROS production and activates antioxidant enzymes to provide cytoprotective benefits against oxidative stress [159].

#### 4.5.5 Antiviral activities

Myricetin has been found effective against the Infectious bronchitis virus (IBV) in chicken embryo kidneys, where the compound remarkably inhibited IBV virus protein named Papain-like protease (PLpro) which is responsible for negative regulation of host innate immune to facilitate viral replication. Moreover, NF-kB and IRF7 signalling pathways, whose transcription levels were downregulated by PLpro, are considerably upregulated by the substance [160].

Myricetin was tested against several HIV-1 viral strains, where it demonstrated more than 80% anti-HIV activity. The most prominent inhibition (more than 90%) was observed against HIV-1 BaL infection. Additionally, myricetin inhibited around 49% HIV-1 reverse transcriptase (RT) enzyme, which is one of the primary targets for various anti-HIV-1 medications in clinical use and plays a crucial role in the HIV life cycle [161].

#### 4.5.6 Cytotoxic activities

Using pull-down, co-immunoprecipitation, and intracellular Ca^2+^ flow tests, myricetin selectively inhibited Moloney murine leukaemia virus-1 (PIM1) and disrupted the link between PIM1 and CXCR4 to have cytotoxic, pro-apoptotic, and anti-metastatic effects on prostate cancer cells [162].

With doses of 5, 25, 50, and 100 μM, myricetin showed cytotoxic action against HCT-15 human colon cancer cells, reducing cell viability in a dose-dependent manner. However, the most notable action was seen at 100 μM of myricetin, which decreased cell viability by 70% when compared to the control group. The treatment also raises the BAX/BCL2 ratio (the ratio of the B-cell lymphoma 2 associated X protein to the B-cell lymphoma 2) but not the cleavage of caspase-3 and -9. The apoptosis-inducing factor was also released from mitochondria, indicating that myricetin causes the death of HCT-15 human colon cancer cells [151].

#### 4.5.7 Lipid-lowering activities

The lipid-lowering activity of myricetin was observed on high-fat diet-induced hypoglycemic rats, where the treatment with a dose of 300 mg/kg/day for 8 weeks notably reduced intercellular accumulation of triglyceride in 3T3-L1 adipocytes, body weight gain, visceral fat-pad weights, plasma lipid levels, hepatic triglycerides hepatic lipid droplets accumulation and epididymal adipocyte size of experimental rats. Also the treatment unregulated expression of PARPα, acyl-CoA oxidase, and cytochrome P450 isoform 4A1, as well as upregulated the hepatic SREBPs expression [163].

In a study, myricetin was shown to have lipid-lowering efficiency in mice fed a high-fat diet. Treatment with doses of 50, 100, and 200 mg/kg body weight of myricetin decreased the levels of TC, TG, LDL, and non-HDL and elevated HDL levels, however, the 50 mg/kg dose had a more pronounced effect than the other two [164].

The multitude of benefits of myricetin indicate its potential as a potent therapy. In medicine, it plays a crucial role as it regulates blood sugar levels, combats inflammation, fights infections, safeguards against harmful molecules, inhibits viral replication, destroys cancer cells, and manages fat metabolism. Particularly noteworthy is its interaction with the GLP-1 receptor, indicating a potential breakthrough in diabetes management. Myricetin's diverse biological activities make it a compelling candidate for further research and development in the fields of diabetes, inflammation, infectious diseases, cancer, and metabolic disorders.

### 4.6 Naringenin

Naringenin (40,5,7-trihydroxy flavanone) chemically known as s 2,3-dihydro-5,7-dihydroxy-2-(4-hydroxyphenyl)- 4H-1-benzopyran-4-one (C_27_H_32_O_14_) with a molecular weight of 580.4 g/mol, is a well-known flavonoid that is largely present in citrus fruits like grapefruits and sour oranges, [[165], [166], [167]]. However, citrus fruits' bitter flavor results from naringin, its common glycoside form [165]. It demonstrated several therapeutic activities including antithrombotic, anti-atherosclerosis, antidiabetic, antihypertension, anti-inflammatory, anti-hyperlipidaemic, and anti-oxidative activities [167].

#### 4.6.1 Antidiabetic activities

A treatment of 4 weeks with 100 mg/kg b. w./day of naringenin improved diabetic markers in nicotinamide/STZ-induced type 2 diabetic rats. This therapy improved the decreasing serum insulin and C-peptide level, the diminished amount of liver glycogen, the raised levels of glucose-6-phosphatase and glycogen phosphorylase in the liver, and the worsened serum lipid profile, glucose transporter type 4 (GLUT4), adiponectin, and insulin receptor b-subunit mRNA levels in adipose tissue were also increased by the treatment [168].

The supplement of 25 mg/kg bw of naringenin effectively reduced diabetic conditions in HFD-fed and STZ-induced diabetic rats. Through modulation of the expression of GLUT-4 and TNF-α, two important pathways related to type 2 diabetic modalities, this study demonstrated that the treatment reduced hyperglycemia and excess insulin levels in diabetic rats. Additionally, this treatment also improved some hyperlipidemic markers and showed potential antioxidant activities in the diabetic group [169].

#### 4.6.2 Antiinflammatory activities

Naringenin exhibited proficient antiinflammatory activity in diabetic rats, where administration of different doses of naringenin decreased renal tumour necrosis factor-α levels and expression in a dose-dependent manner. It has also been observed that the production and expression of IL-1β, IL-6, monocyte chemoattractant protein-1, type IV collagen, fibronectin, transforming growth factor-β1, protein kinase C activity, and NF-κB p65 and p50 were significantly decreased by naringenin [170].

In a different trial, 30 days of therapy with 50 mg/kg/bw/day of naringenin prominently decreased the elevated levels of some inflammatory markers including serum aspartate and alanine transaminases, iron, ferritin, TNF-α, IL-6, NF-κB, COX-2, MIP-2, CD14 and iNOS protein adducts in the ethanol-induced injured liver of experimental rats [171].

#### 4.6.3 Antimicrobial activities

Naringenin was found to limit *Streptococcus mutans* progress and biofilm formation, enhance the hydrophobicity of the surface of *S. mutans*, decrease bacterial aggregation, and downregulate the mRNA expression of glycosyltransferases B, glycosyltransferases C, comD, comE, and luxS genes related to S.mutans growth and biofilm formation [172].

Naringenin and its named 7-O-butyl naringenin exhibited potential antimicrobial activity against different strains of *H. pylori*. Concentration range from 5 to 20 μM of naringenin manifested a strong zone of inhibition against 26 695, 51, and SS1 strains *H. pylori*, while 20 μM concentration of the derivative exerted a moderate zone of inhibition against these strains [173].

#### 4.6.4 Antioxidant activities

Naringenin revealed enhanced effectiveness in preventing oxidative damage to lipids in a dose-dependent manner. The IC_50_ value was observed at 21.00 ± 0.016 and 0.23 ± 0.003 mmol/l respectively for peroxyl and hydroxyl radicals scavenging [174].

Also, research showed that naringenin suppresses the free radical-mediated neurotoxicity associated with the amyloid β protein, which is one of the key hypotheses for the origin of Alzheimer's disease [65].

#### 4.6.5 Antiviral activities

A study demonstrated the antiviral effect of naringenin, where 52.64 μg/mL of the compound exhibited virucidal activity against 50% of Vero cells infected with Dengue virus type-2 [175].

In another research antiviral effect of naringenin was found against Hepatitis C virus (HCV), where it dose-dependently inhibits HCV production without affecting intracellular viral RNA or protein levels. The halting of viral assembly, which prevents the generation of contagious viral particles, was the cause of the antiviral effect. The activation of PPARα played a role in this inhibition, which decreased the generation of VLDL without increasing hepatic lipid buildup. Similar to interferon therapy, long-term naringenin administration caused a rapid 1.4 log drop in HCV levels [176].

However, naringenin conveyed dose-dependent inhibitory activity against BHK-21 and Vero's cells infected with the chikungunya virus. The substance prevented chikungunya virus multiplication in its post-entry phases with an IC_50_ of 6.818 μM [126].

#### 4.6.6 Cytotoxic activities

Cytotoxicity against the human breast cancer model (mammosphere) was observed by naringenin. The medication prevented the creation of mammospheres and colonies from MCF-7 cells as well as migration and the transition from epithelial to mesenchymal in the mammosphere. Furthermore, 200 and 100 μM drug concentrations, respectively, were shown to provide more than 80% cell viability during colony and mammosphere formation [177].

By the inhibition of mitogen-activated protein kinase in MCF-7 breast cancer cells, naringenin reduces cell growth and causes apoptosis in estrogen-resistant breast cancer. Moreover, the substance reduced the survival and cell proliferation of MCF-7 breast cancer cells that were resistant to tamoxifen (antagonist of estrogen receptor) as well as confined estrogen receptor alpha to the perinuclear region of the cancerous cell [178].

#### 4.6.7 Lipid-lowering activities

Treatment with a 25 mg/kg bw dose of naringenin for 45 days suppressed the elevated hyperlipidemic markers including TC, TG, LDL, VLDL, and FFA levels in HFD-fed and STZ-induced hyperlipidemic diabetic rats, while elevated HDL levels which were decreased in diabetic rats [169].

Similarly, in STZ-induced type 2 diabetic rats, 4 weeks of treatment with 100 mg/kg b. w./day with naringenin decreased the high TC, TG, LDL, VLDL, and FFA levels and increased the lowered HDL level [168].

In short, naringenin is a natural compound found in citrus fruits that has the ability to help treat diabetes and its related problems. By helping with diabetic markers, regulating glucose metabolism enzymes, improving insulin sensitivity, and reducing inflammation, it effectively helps in controlling diabetes. Furthermore, it has abilities to fight against bacteria and viruses and can also help prevent damage from harmful substances in the body. This makes it a potentially beneficial natural substance for treating different illnesses, such as cancer. The multifaceted benefits of naringenin underscore its potential as a valuable asset in the management of diabetes and related health issues.

### 4.7 Quercetin

The phytochemical quercetin (3,5,7-trihydroxy-2-(3,4-dihydroxy phenyl)-4Hchromen-4-one), a dietary flavonoid with the chemical formula C_15_H_10_O_7_ and the molecular weight 302.236 g/mol is found in a variety of foods, including capers, black chokeberries, onions, tomatoes, and lettuce [179,180]. Quercetin typically exists in a bonded form with 69 ethers, phenolic acids, and other substances in a plant. Different quercetin derivatives appear to 70 affects how quickly they are absorbed in the abdomen and small intestine [181,182]. According to some reports, quercetin is a bioactive chemical with anti-inflammatory, antioxidant, hypertensive, anti-obesity, anti-hypercholesterolemic, anti-atherosclerotic, and antitumor activities [183].

#### 4.7.1 Antidiabetic activities

When administered of quercetin for six weeks at a dose of 10 mg/kg body weight, quercetin significantly reduced blood sugar levels in STZ-induced diabetic rats. It also improved responses to the a1-adrenoceptor agonist phenylephrine (PE) and restored impaired relaxations to the endothelium-dependent vasodilator acetylcholine (ACh) [184].

In a randomized, double-blind, placebo-controlled parallel study, quercetin was tested on 84 women with an endocrine gland problem called polycystic ovary syndrome, which has multiple causes and is genetically complex, here quercetin partially increases the level of adiponectin by 5.56% as well as high molecular weight adiponectin by 3.9% as compared to the placebo, the resulting improvement in adiponectin-mediated insulin resistance [185].

Additionally, in STZ-induced diabetic rats, a 21-day therapy with 100 mg/kg oral quercetin and 1% topical quercetin significantly depressed blood glucose levels [168].

#### 4.7.2 Anti-inflammatory activities

In a randomized, double-blinded, placebo-controlled study, quercetin showed potential anti-inflammatory activity in 8 nonsmokers with untreated sarcoidosis; their mean age was 45,00 ± 10.00 years. The treatment significantly decreased the basal and *ex vivo* LPS-induced levels of the two inflammatory markers TNFα/IL-10 and IL-8/IL-10 [186].

Another randomized double-blind clinical trial with 60 participants (21.00 ± 1.60 years aged) showed the anti-inflammatory activity of quercetin, where treatment with 500 mg of quercetin for 8 weeks expressively reduced inflammatory biomarkers namely plasma C-reactive protein (CRP), IL-6 but the effect was not notable for E-selectin. However, in the same study most prominent activity was observed by the combined supplement of quercetin and vitamin C [187].

#### 4.7.3 Antimicrobial activities

In a study, the antibacterial activities of quercetin were tested against *S. aureus, E. coli, Shigella flexneri, Proteus vulgaris, P. aeruginosa,* and *Lactobacillus casei* var *shirota* by broth dilution method, interestingly most potent activity was observed against *S. aureus* and *P. aeruginosa* with MIC of 20 mcg/mL, while median activity was observed against *P. vulgaris* and *E. coli* by 300 mcg/mL and 400 mcg/mL MIC respectively. In contrast, no activity was found against *S. flexneri* and *L. casei* var *shirota* at the highest concentration of 500 mcg/mL [188].

Another study found that quercetin significantly reduced the growth of the filamentous fungus *Aspergillus flavus*, the gram-positive bacteria *Sarcina maxima* and *Micrococcus kristinae*, and the Gram-negative bacteria *Klebsiella pneumoniae* [189].

#### 4.7.4 Antioxidant activities

Quercetin (10 mg/kg b.w.) was administered to STZ-induced diabetic rats in a study, and it was discovered that the treatment reduced the levels of plasma malondialdehyde (MDA) and 4-hydroxyalkyne (4-HNE) in the diabetic rats while elevating superoxide dismutase activity and total antioxidant capacity [184].

Through a randomized, double-blinded, placebo-controlled trial, quercetin showed potential antioxidant activity in 8 nonsmokers with untreated sarcoidosis, whose mean age was 45.00 ± 10.00 years. Treatment with 4 × 500 mg/day of quercetin for 24 h improved the antioxidant defence, as shown by the amplified total plasma antioxidant capacity as well as the lowered levels of inflammation and oxidative stress indicators in the blood of sarcoidosis patients [186].

#### 4.7.5 Antiviral activities

In a randomized, open-label, controlled clinical study that lasted two weeks, 42 COVID-19 outpatients were given quercetin to see if it had any antiviral effects against the SARS-CoV-2. Interestingly, a dose of 1500 mg per day for one week followed by 1000 mg per day for another week significantly increased the virus's ability to clear the body of it, decreasing the occurrence of symptoms. Also, the treatment improved the disease biomarkers by reducing lactate dehydrogenase, ferritin, lactate dehydrogenase, and D-dimer by 35.5%, 40%, 54.8%, and 11.9% respectively [190].

In another study, quercetin manifested a potential antiviral effect against Influenza A Viruses (IAVs), where it inhibited influenza infection with a variety of strains, including A/Puerto Rico/8/34 (H1N1), A/FM-1/47/1 (H1N1), and A/Aichi/2/68 (H3N2). The IC_50_ values for quercetin against these strains were 7.756 ± 1.097, 6.225 ± 0.467, and 2.738 ± 1.931 μg/mL, respectively [191].

#### 4.7.6 Cytotoxic activities

Quercetin manifested cytotoxic activity on two human colonic cancer cell lines, HT29, and HCT15 with IC_50_ values of 42.50 μM and 77.40 μM, respectively. However, 40 μM quercetin in both cell lines boosted NFkB's nuclear translocation. However, 40 μM quercetin activated caspase-3, elevated cytosolic cytochrome *c*, reduced pAkt, pGSK-3b, and cyclin D1 levels, and overexpressed COX-2 in HT29 cells [192].

It was observed by another study that, quercetin inhibits cancer cell proliferation by interfering with tubulin and perturbing microtubule functions. Additionally, it binds to tubulin at a specific site, induces conformational changes, and disrupts microtubulate polymerization dynamics, ultimately leading to the inhibition of cancer cell growth [152].

#### 4.7.7 Lipid-lowering activities

Quercetin conveyed potential hypolipidemic activity in STZ-induced diabetic rats, where a 21 days treatment with 100 mg/kg oral quercetin along with 1% topical quercetin prominently reduced TC, TG, and LDL levels with values of 68.59 ± 2.04, 44.68 ± 1.18, and 23.97 ± 2.10 mg/dL respectively compared to diabetic control 83.02 ± 2.98, 72.54 ± 2.83 and 47.55 ± 3.24 mg/dL respectively. However, the treatment increased plasma HDL, Total plasma proteins, and albumin levels with values of 35.68 ± 1.25 mg/dL, 5.968 ± 0.145 and 5.06 ± 0.31 g/dl respectively compared to the diabetic control group 20.97 ± 2.14 mg/dL, 5.008 ± 0.254 and 4.05 ± 0.10 g/dl respectively [193].

In another study, at 10 μmol/L of polyphenols and 100 μmol/L of polyphenols, quercetin significantly reduced the cholesterol concentration in erythrocytes to 75% and 69%, respectively, of starting values. Even more severe reductions were observed in isolated erythrocytes, reaching 70% at 10 μmol/L and 58% at 100 μmol/L of polyphenols, respectively, of baseline values [194].

To summerize, quercetin, a potent flavonoid abundant in plant-based foods, showcases substantial therapeutic potential spanning diverse health conditions. The fact that it can lower blood sugar levels, improve blood vessel function, and reduce inflammation in both rats and humans with diabetes shows that it has properties that help with diabetes and inflammation. Additionally, its broad-spectrum antimicrobial effects against bacteria and fungi, coupled with its antioxidant capabilities, highlight its versatile nature. Quercetin's promising antiviral activity against SARS-CoV-2 and Influenza A viruses, along with its cytotoxicity against cancer cells and lipid-lowering effects, further emphasize its role as a natural therapeutic agent. These multifaceted properties position quercetin as a valuable option in addressing conditions ranging from diabetes and inflammation to antimicrobial and anticancer applications.

## 5 Discussion

Over the past ten years, flavonoids have attracted a lot of interest in the literature, and a number of potential positive benefits have been clarified [12]. Due to chemical composition and structural analogies with various biomolecules and their crucial role in plant-insect and plant-bacterial interactions, flavonoids are a desirable class of phytoconstituents for biological activity. They make suitable chemical scaffolding for novel therapeutic medicines because of their extensive occurrence, wide range diversity, and natural origin [195]. Recent studies on the health advantages of flavonoids have concentrated on eating habits, theoretical studies examining the mechanisms by which flavonoids function in people, including and integrating information from basic research, *in vitro* and *in vivo* studies, and links between particular flavonoids and particular health conditions [196].

Moreover, they have been said to have anti-inflammatory, antioxidant, antibacterial, antiviral, antiallergic, cytotoxic, and anticancer activities. They are also used to treat neurological illnesses and have a vasodilatory effect. Additionally, flavonoids have been shown to reduce the enzyme activity of cyclooxygenase and lipoxygenase, lipid peroxidation, platelet aggregation, capillary permeability, and fragility. However, they block a wide range of enzymes, including hydrolases, hyaluronidase, ALP, arylsulphatase, cAMP phosphodiesterase, lipase, and alpha-glucosidase kinase [15]. Also, it has been established *in vitro* and *in vivo* models that flavonoids can inhibit steroid-hormone-dependent cancers through a number of different mechanisms. Among other things, they have qualities that control steroid hormones, limit proliferative growth, and fight cancer [196].

**Fig. 2:**
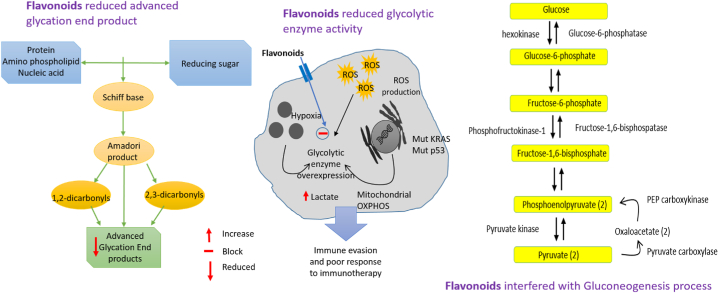
Antidiabetic effect of flavonoids results from glycation, gluconeogenesis, and glycolytic enzyme activity. Flavonoids reduce the glucose level in the body by interfering with gluconeogenesis. Also, flavonoids reduce advanced glycolation end products along with glycolytic enzyme overexpression; Reactive oxygen species (ROS).

**Fig. 3:**
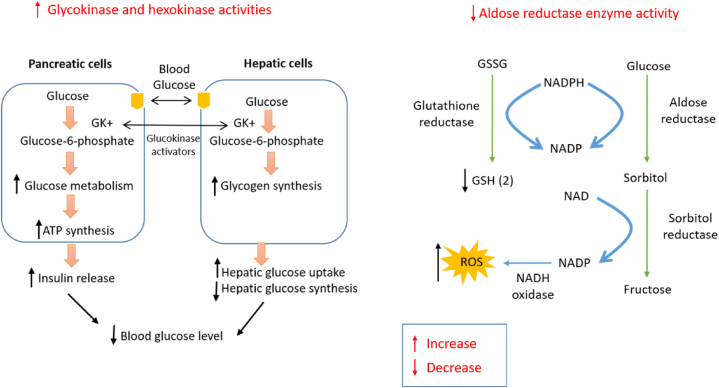
Antidiabetic effect of flavonoids involving glucokinase, hexokinase, and aldose reductase enzyme activities.Flavonoids reduce blood glucose level by reducing glycokinase and hexokinase activities. They also interfere with aldose reductase actvity which hampered fructose production from glucose; glutathione disulfide (GSSG); glutathione (GSH); nicotinamide adenine dinucleotide (NAD); nicotinamide adenine dinucleotide phosphate (NADP); nicotinamide adenine dinucleotide phosphate hydrogen (NADPH).

Flavonoids like apigenin, galangin, hesperetin, kaempferol, myricetin, naringenin, and quercetin demonstrated diverse pharmacological activities in *in vitro*, *in vivo*, and *in silico* studies. Fig. 2, Fig. 3 illustrated that these compounds exhibited antidiabetic effects through various mechanisms involving the reduction of aldose reductase enzyme [86,197], gluconeogenesis [134,198], and insulin receptor expression and resistance [135,199].

**Fig. 4:**
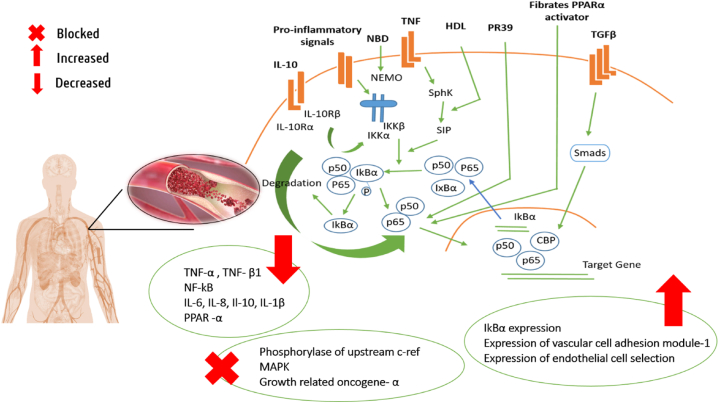
Anti-inflammatory effect of flavonoids through reduction of inflammatory markers, supression of inflammatory genes, and increasing expression of antiinflammatory genes. Flavonoids prohibit growth related oncogene and phosphorylase enzyme responsible for inflammation, increase expression of inflammatory suppressive genes like IkBα, vascular cell adhesion module-1, endothelial cell resulting in decreased antiinflammatory markers like tumer necrosis factor (TNF), Interleukin (IL), peroxisome proliferator-activated receptors (PPARs).

Consequently, they increase glucokinase and hexokinase activities [33,200] and GLUT-3 and GLUT-4 activities [88,168]. The antiinflammatory effect of these seven flavonoids demonstrate in Fig. 4 where they decreased TNF-α, TNF- β1, NF-kB, IL-6, IL-8, Il-10, IL-1β, PPAR-α [90,201], inhibited phosphorylase of upstream c-ref, MAPK, and growth related oncogene-α [129], and increased expression of IkBα [91], vascular cell adhesion module-1, and endothelial cell selection [138].

**Fig. 5:**
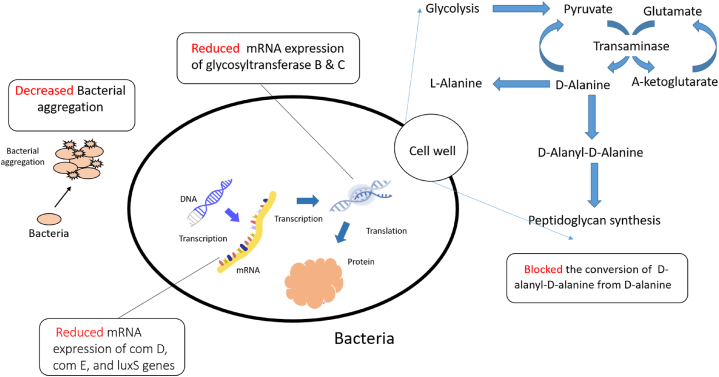
Antibacterial effect of flavonoids by interfearing bacterial central dogma as well as cell wall synthesis. Flavonoids reduce mRNA expression of glycotransferase which hampers bacterial translation process. Flavonoids also interfere with bacterial mRNA to block bacterial transcription process and arrest cell wall synthesis by blocking conversion of d-alanine.

**Fig. 6:**
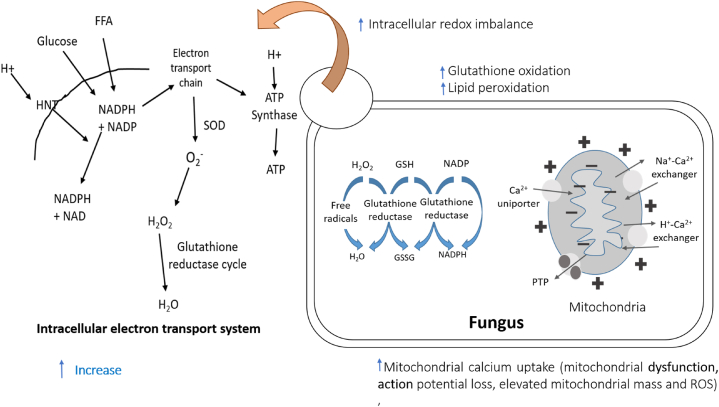
Antifungal effect of flavonoids involves fungal intracellular redox imbalance, free radical activity, and mitochondrial ion exchange. Flavonoids interfere with the fungal intracellular electron transport system by overexpressing the glutathione reductase enzyme, which inhibits the production of lethal hydrogen peroxide. Flavonoids are also capable of creating fungal mitochondrial dysfunction by increasing mitochondrial calcium uptake: Nicotinamide adenine dinucleotide phosphate (NADP); Nicotinamide adenine dinucleotide phosphate hydrogen (NADPH); Glutathione (GSH); Glutathione disulfide (GSSH), Adenosine triphosphate (ATP), Free fatty acids (FFA).

These compounds also showed various mechanisms of action related to their antimicrobial action like increasing intracellular redox imbalance [18,202], glutathione oxidation, lipid peroxidation mitochondrial calcium uptake [18] of fungus as well as reducing the bacterial aggregation, mRNA expression of com D, com E, and luxS genes, and glycosyltransferase B & C [172], blocking the d-alanine pathway of bacteria [83,203] illustrated at Fig. 5, Fig. 6.

**Fig. 7:**
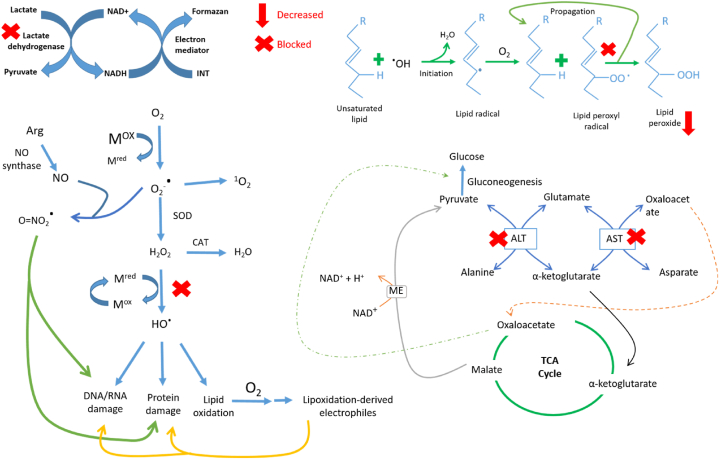
Antioxidant effect of flavonoids by reducing free radical-mediated neurotoxicity and lipid peroxidation. Flavonoids block the conversion of free radicals, resulting in protection from frradical-mediated cell damage. Moreover, flavonoids can block lactate dehydrogenase, which is responsible for the conversion of pyruvate from lactate, in relation to the oxidative reactions: Aspartate transaminase (AST); Alkaline phosphatase (ALP); Alanine transmitase (ALT); Tricarboxylic acid cycle (TCA).

However, Fig. 7 implied that various mechanisms including reducing free radical-mediated neurotoxicity [65,204], ALP, ALT, ASP [92,205], lipid peroxidation [93,206], lactate dehydrogenase [190,207] along with inhibiting the activation of T-cell [143], these flavonoids exhibited promising antioxidants activity. (2)

**Fig. 8:**
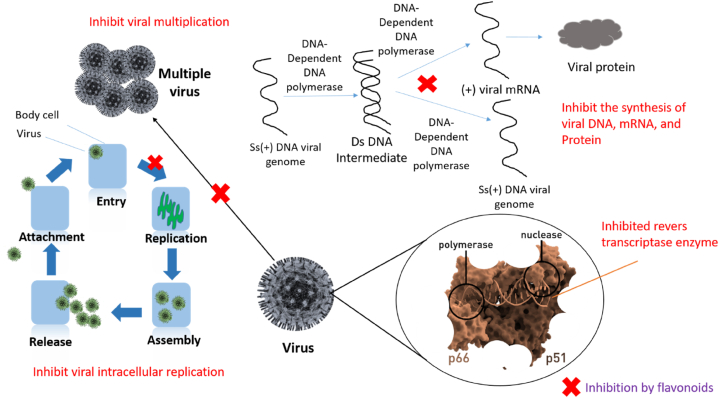
Antiviral effect of flavonoids by interfearing viral protein synthesis as well as viral replication. Flavonoids inhibit the intracellular replication of virus along with reverse transcriptase enzyme which is responsible for viral multiplication. They can also hamper central dogma of virus.

Moreover, by inhibiting viral multiplication through ERK/MNK1/eIF4E signaling pathway [94], synthesis of viral DNA, mRNA, and protein [94,208], revers transcriptase enzyme [161], and viral intracellular replication [126] while increasing innate immune-related genes expression [127] these flavonoids showed potential antiviral effect illustrated on Fig. 8.

**Fig. 9:**
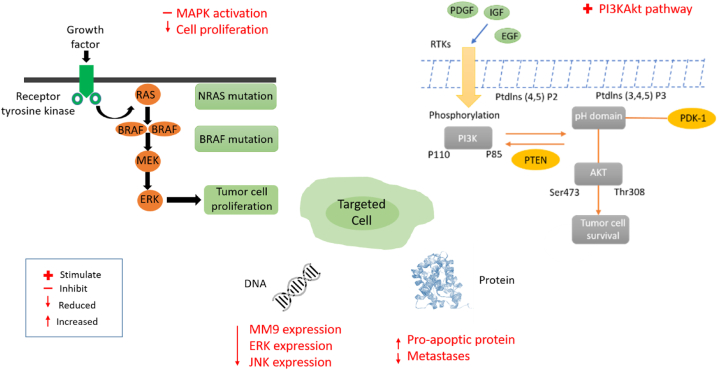
Cytotoxic effect of flavonoids related to MAPK and PI3KAkt pathways. Flavonoids reduced cell proliferation by blocking Mitogen-activated protein kinase (MAPK). They are also capable to stimulate PI3KAkt expression which reduced tumer cell survival. Besides, flavonoids result in reduction of metastases by increasing pro-apoptic protein.

**Fig. 10:**
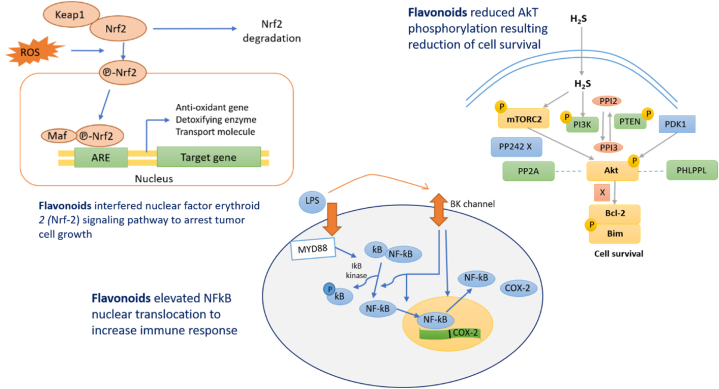
Cytotoxic effect of flavonoids resulted from nuclear factor erythroid 2 (Nerf-2), Ak strain transforming (Akt), and nuclear factor kappa B (NFƙB) pathways. Flavonoids interfere with Nrf-2 signaling pathway to arrest tumor cell growth, increase immune response by elevating nuclear translocation of nuclear factor kappa B (NFƙB) and reduce cell survival by decreasing Akt phosphorylation; cyclooxygenase-2 (COX-2).

The cytotoxic effects of these compounds were observed through different mechanisms like stimulation of PI3KAkt pathway [118,209], NFkB nuclear translocation [153,192], reduction of cell proliferation [96,210], expression of MM9, ERK, JNK [100], metastases [97], and AkT posphorylation [114,211]. However, flavonoids block MAPK activation [129,210] as well as the Nrf-2 signaling pathway [116,212] (Fig. 9, Fig. 10).

**Fig. 11:**
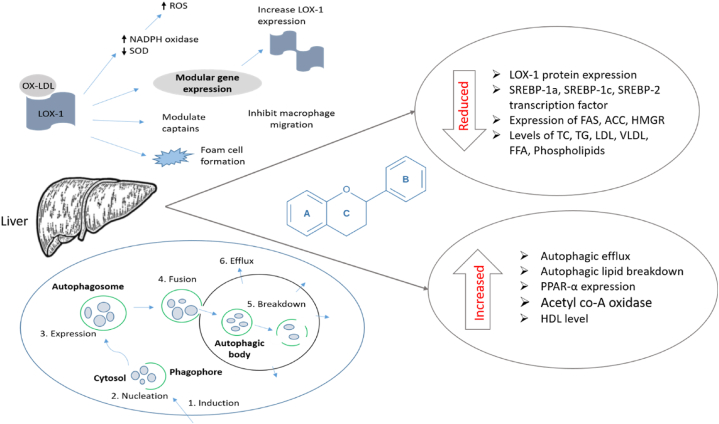
Lipid-lowering effect of flavonoids hindering autophagic and LOX-related pathways. Flavonoids interfere with LOX-1 (Lectin-like oxidized low density lipoprotein receptor 1) resulting in reduction of low density lipoprotein. They also increase activity of autophagic process which helps to inhibit fat cells.

Last but not least, the lipid-lowering activity of these compounds was observed through various mechanisms including the reduction of LOX-1 protein expression [84,213], SREBP-1a, SREBP-1c, SREBP-2 transcription factor, expression of FAS, ACC, HMGR [117], and levels of TC, TG, LDL, VLDL, FFA, phospholipids [33,119]; while increasing autophagic efflux [99,214], autophagic lipid breakdown [99], PPAR-α expression, acetyl Co-A oxidase and HDL levels [148] (Fig. 11).

Both of these substances have been shown to have the aforementioned properties, particularly strong antioxidant potency. Despite the fact that all of these flavonoids have antimicrobial properties, apigenin stood out for having activity against a variety of bacteria and fungi [16]. Hesperetin, however, has shown extremely significant antifungal action, particularly against the *Candida* genus [124]. These chemicals' potent anti-inflammatory effects are primarily attributed to the inhibition of various inflammatory markers, including TNF-α, NF-κB, IL-1β, IL-6, IL-8, and others which are mentioned on these article. With the exception of galangin, they all demonstrated highly effective antiviral activity against several infections. Clinical investigations specifically showed that kaempferol and quercetin have an antiviral effect against the SARS-COV-2 virus, which was the cause of the recent pandemic scenario [146]. Antidiabetic activities of these compounds were noteworthy, all of them was found to have strong antidiabetic activities, especially by decreasing glucose level, and insulin resistance as well as improving other diabetic parameters. Also in a clinical study, quercetin showed antidiabetic potential [185]. The cytotoxic activity of flavonoids is well-known, and it was evident in this review [15]. Additionally, these seven flavonoids greatly enhanced hyperlipidemic conditions.

## 6 Novelty of this review work

Flavonoids are the most reached and discussed phytochemical class for the deviation of lead compounds in the drug discovery process [215]. Our manuscript presents a comprehensive review of the most notable dietary flavonoids and their diverse pharmacological actions, making them an attractive target for lead compound discovery in the drug development process. Moreover, the paper includes schematic representations of the mechanisms through which these flavonoids exert their pharmacological potential, providing valuable insights for further research. To the best of our knowledge, no previous review has covered these seven potent dietary flavonoids in such detail or with accompanying schematic sketches.

## 7 Limitations

For the pharmaceutical research community, the process of drug discovery and development presents substantial difficulties. The ADME procedure, which examines how a medication molecule behaves after consumption, is one of the most important ones [216]. Drug metabolism studies are essential for identifying active metabolites for new chemical entities, identifying reactive or toxic metabolites, mitigating safety concerns, ensuring the suitability of animal models for human metabolism, and facilitating precise dose estimations in humans [217]. However, the ADMET and PK/PD processes of these flavonoids are not well established. Pre-human testing toxicity has been responsible for a substantial number of compound failures at the clinical stage of drug development, accounting for up to 30% of losses. Toxicology studies including at least two nonhuman species are frequently used to determine a safe dose range and acquire an understanding of drug distribution, organ-specific toxicity, and metabolism [218]. These research projects are essential for spotting potential safety concerns and directing future growth. The toxicological properties of these seven flavonoids have not been fully elucidated. Moreover, the specific dosages of these compounds and safety data regarding their use for lactating mothers, children, and the elderly have not been thoroughly investigated yet.

## 8 Significance and practical implications of this study

Exploring the various bioactive characteristics of these substances offers a viable path toward their possible application as natural phytoconstituents in disease management. Contrarily, synthetic medications have a number of disadvantages, such as low bioavailability, problems with cost-effectiveness, drug resistance, and the potential for unanticipated negative consequences [219]. This emphasizes the potential benefits of using natural chemicals' medicinal potential for better healthcare solutions. For millennia, plants have bestowed us with essential phytochemicals, serving as invaluable resources [220]. These bioactive compounds are crucial in drug research and development, with approximately 80% of existing medications either originating directly from plants or being derivatives of them [216]. Therefore, these substances provide great promise as potential future natural medication candidates. Extensive research is essential to realize their full potential. This entails researching and improving important elements including pharmacokinetic/pharmacodynamic characteristics, therapeutic index, safety and toxicological profiles, dosage calculation, medication interactions (both with other pharmaceuticals and food), and other critical variables. To establish these compounds as innovative medications or lead compounds, a thorough examination and analysis of these characteristics is required. This review seeks to provide useful guidance for upcoming academics performing additional research on these exceptional flavonoids.

## 9 Conclusion and future recommendations

Since the majority of people are exposed to flavonoids on a regular basis, their effect on human health is significant. The current situation reveals that multiple experimental investigations of the diverse biological activities of flavonoids have been conducted, but these findings have not been compiled into a single comprehensive article. Seven dietary flavonoids including apigenin, galangin, hesperetin, kaempferol, myricetin, naringenin, and quercetin have shown significant potential in diverse health-related activities. These include benefits such as antidiabetic, anti-inflammatory, cytotoxic, antimicrobial, antiviral, antioxidant, and lipid-lowering effects. These findings are based on a comprehensive analysis of data obtained from *in vitro, in vivo,* and clinical studies. The diverse range of activities exhibited by these flavonoids underscores their significance in therapeutic applications and health promotion. Even while our research sheds light on the various bioactivities of these flavonoids, it is important to recognize that some elements, such as various pharmacokinetic/pharmacodynamic parameters and toxicological studies, have not yet been fully explored. Therefore, more thorough research is required to comprehend the processes underlying the actions of these flavonoids and their potential consequences on human health. Despite these restrictions, this review work offers important new information on the possible health advantages of these flavonoids. Their actions across various health areas have shown promise for the creation of new therapeutics and functional food additives. In conclusion, this research lays the groundwork for future research into the therapeutic potential of these flavonoids, highlighting the necessity of further, in-depth research to fully harness their positive impacts on human health. Such initiatives will speed up the creation of interventions that are supported by data and improve our knowledge of the complex mechanisms underlying the bioactivities of flavonoids. In the end, this knowledge might result in enhanced medical interventions and disease-prevention plans that would help people all around the world.

## Declarations

All concerned authors have read the manuscript and given their approval for submission. No portion of the article has ever been published before, and no portion of it is presently being considered for publication in any journal.

## Data availability statement

Data included in article/supplementary material/referenced in article.

## CRediT authorship contribution statement

**Hasin Hasnat:** Writing – original draft, Methodology, Formal analysis, Data curation, Conceptualization. **Suriya Akter Shompa:** Writing – original draft, Methodology, Investigation, Data curation, Conceptualization. **Md. Mirazul Islam:** Software, Resources, Investigation, Formal analysis, Data curation. **Safaet Alam:** Writing – review & editing, Visualization, Validation, Supervision, Project administration, Investigation, Formal analysis. **Fahmida Tasnim Richi:** Writing – original draft, Resources, Methodology, Investigation, Formal analysis, Data curation. **Nazim Uddin Emon:** Writing – review & editing, Visualization, Validation, Project administration, Formal analysis. **Sania Ashrafi:** Writing – review & editing, Visualization, Validation, Resources, Methodology. **Nazim Uddin Ahmed:** Visualization, Software, Methodology, Formal analysis, Data curation. **Md. Nafees Rahman Chowdhury:** Writing – review & editing, Visualization, Validation, Resources, Project administration. **Nour Fatema:** Writing – review & editing, Visualization, Validation, Formal analysis, Data curation. **Md. Sakhawat Hossain:** Resources, Methodology, Formal analysis. **Avoy Ghosh:** Software, Methodology, Formal analysis, Data curation. **Firoj Ahmed:** Writing – review & editing, Visualization, Validation, Supervision, Resources, Project administration.

## Declaration of competing interest

The authors declare that they have no known competing financial interests or personal relationships that could have appeared to influence the work reported in this paper.
